# Lipid Droplet‐Organized MDM2‐Mediated P53 Degradation: A Metabolic Switch Governing Diet‐Driven Tumor Progression

**DOI:** 10.1002/advs.202503473

**Published:** 2025-06-05

**Authors:** Haiyang Liu, Lin Jing, Yixin Li, Jinxing Zhou, Xiaohui Cui, Sen Li, Shijie Yang, Fangming Kan, Junfeng Du, Wentao Zhong, Sheng Yu, Ning Wang, Xing Jia, Junhui Li, Pan Nie, Zhenzhong Chen, Ying Han, Lingxi Jiang, Xiyun Yan, Hongxia Duan, Baiyong Shen

**Affiliations:** ^1^ Department of General Surgery Pancreatic Disease Center, Ruijin Hospital Shanghai Jiao Tong University School of Medicine Shanghai 200025 China; ^2^ Research Institute of Pancreatic Diseases Shanghai Key Laboratory of Translational Research for Pancreatic Neoplasms Shanghai Jiao Tong University School of Medicine Shanghai 200025 China; ^3^ State Key Laboratory of Systems Medicine for Cancer Institute of Translational Medicine Shanghai Jiao Tong University Shanghai 200025 China; ^4^ CAS Engineering Laboratory for Nanozyme Key Laboratory of Biomacromolecules (CAS) CAS Center for Excellence in Biomacromolecules Institute of Biophysics Chinese Academy of Sciences Beijing 100101 PR China; ^5^ Nanozyme Laboratory in Zhongyuan Henan Academy of Innovations in Medical Science Zhengzhou 451163 China; ^6^ University of Chinese Academy of Sciences Beijing 100049 China; ^7^ National Laboratory of Macromolecules Institute of Biophysics Chinese Academy of Sciences Beijing 100101 China; ^8^ National Institute of Biological Sciences Beijing 102206 China; ^9^ School of Information Science and Technology Beijing Forestry University Beijing 100083 China; ^10^ Engineering Research Center for Forestry‐oriented Intelligent Information Processing of National Forestry and Grassland Administration Beijing 100083 China; ^11^ Department of Biochemistry & Immunology Capital Institute of Pediatrics Beijing 100020 China; ^12^ Medical Department of General Surgery The 1st Medical Center Chinese PLA General Hospital Beijing 100853 China; ^13^ Department of General Surgery The 7th Medical Center Chinese PLA General Hospital Beijing 100700 China; ^14^ The Second School of Clinical Medicine Southern Medical University Guangdong 510515 China; ^15^ The Second School of Clinical Medicine Southern Medical University Guangzhou 510515 China; ^16^ Center for Biological Imaging, Core Facilities for Protein Science, Institute of Biophysics CAS Beijing 100101 China

**Keywords:** high‐fat diets, lipid droplets, p53 degradation initiation, tumor growth

## Abstract

TP53 inactivation in human cancers often results from MDM2/MDMX overexpression, yet therapeutic targeting remains challenging owing to incomplete mechanistic understanding. Lipid droplet (LD) enrichment is identified as a key trigger for MDM2‐mediated p53 degradation. High‐fat diet (HFD)‐induced LD accumulation in tumor cells elevates LD‐surface MDM2 through Cyb5r3‐Myh9 interactions, which recruit cytoplasmic p53/Myh9 complexes to LDs. This spatial proximity enhances MDM2‐p53 binding, accelerating its ubiquitination and proteasomal degradation. Degraded p53 releases the RPS3A‐C/EBPβ complex, upregulating LD‐promoting factors such as CD36 to establish a cell‐autonomous feed‐forward loop. Critically, pharmacological LD reduction (via lipogenesis inhibitors) or switching of tumor‐bearing mice from an HFD to a normal diet restores p53 levels and suppresses tumor growth. These findings delineate a lipid‐driven regulatory axis in which LD biogenesis initiates MDM2‐dependent p53 destruction, reshaping tumor cell lipid metabolism. This mechanism links dietary lipids to oncogenesis through organelle‐specific protein trafficking and provides a therapeutic rationale for targeting lipid metabolism in tumors. This study resolves critical gaps in p53 regulation while proposing dual intervention strategies: disrupting LD‐MDM2 colocalization and modulating lipid availability.

## Graphical abstract

Tumor‐associated TP53 inactivation correlates with lipid droplet (LD) accumulation. High‐fat diets drive Cyb5r3‐Myh9‐mediated p53 enrichment on LD surfaces, accelerating its degradation while upregulating LD‐promoting factor CD36 expression to establish a feed‐forward loop. LD suppression or dietary intervention restores p53 and inhibits tumorigenesis, unveiling LD‐driven p53 regulation mechanisms and novel lipid‐metabolism‐targeted therapeutic strategies.

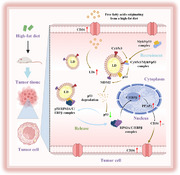

## 1 Introduction

P53, known as the “guardian of the genome,” regulates cell growth, DNA repair, and apoptosis. P53 prevents cancer by monitoring DNA for damage and initiating repair, cell cycle arrest, or cell death.^[^ ^1^ ^]^ However, in the majority of human cancers, the p53 gene is mutated or inactivated, leading to a loss of its tumor suppressive functions.^[^ ^1^ ^]^ The inactivation of p53 is a critical event in the development of many types of cancer, including lung, breast, colon, and brain cancers.^[^ ^2^ ^]^ p53 is inactivated in cancer through a variety of mechanisms, including gene mutations, protein–protein interactions, disruption of regulatory pathways, and other complex mechanisms.^[^ ^2^ ^]^ Restoring p53 function or targeting the pathways that regulate p53 have therefore become important strategies in cancer therapy and drug development.^[^ ^3^ ^]^

MDM2 is recognized as one of the most potent apoptosis inhibitors discovered to date, and is closely correlated with malignant tumors.^[^ ^4^ ^]^ Acting as a negative regulator of p53, MDM2 primarily modulates the function of p53 through multiple mechanisms.^[^ ^5^ ^]^ First, MDM2 directly inhibits the N‐terminal transcriptional activation domain of p53, thereby suppressing its transcriptional activity.^[^ ^6^ ^]^ Second, MDM2 facilitates the nuclear‐to‐cytoplasmic translocation of p53, further reducing the tumor‐suppressive effects of p53 within the nucleus.^[^ ^7^ ^]^ Finally, as an E3 ligase, MDM2 promotes the ubiquitination of p53, leading to its degradation by proteasomes and the complete abrogation of its tumor‐suppressive function.^[^ ^8^ ^]^ Blocking the MDM2‐p53 pathway has emerged as an effective strategy for discovering and developing potent inhibitors of tumor growth.^[^ ^9^, ^10^ ^]^ Despite the relatively deep understanding of the process of MDM2‐mediated p53 degradation, how tumor cells initiate this process remains unknown.

Growing evidence suggests that dietary fat increases solid tumor growth and metastasis^[^ ^11^, ^12^ ^]^ and enhances the abundance of lipid droplets in tumor cells.^[^ ^13^ ^]^ However, gaps exist in our understanding of the relationship between diet and tumor growth. Increasing evidence has shown that lipid droplets (LDs; also known as lipid bodies), which are lipid‐rich cytoplasmic organelles with important functions in intracellular lipid storage, play a crucial role in promoting tumor development, significantly enhancing key processes such as tumor occurrence, development, and metastasis.^[^ ^14^ ^]^ Lipid synthesis, uptake, and breakdown processes are altered in tumor cells, leading to the accumulation of LDs within the cell.^[^ ^15^ ^]^ These accumulated LDs not only provide abundant energy for tumor cells but also contribute to maintaining cancer cell stemness.^[^ ^16^ ^]^ Intracellular crosstalk between LDs and protumor signaling pathways has been reported to determine tumorigenesis.^[^ ^17^, ^18^ ^]^ Therefore, exploring the mechanisms of the intracellular crosstalk of LDs and regulatory pathways in tumorigenesis can not only help us gain a more comprehensive understanding of tumor occurrence and development but also identify new approaches for tumor diagnosis, treatment, and prevention.

Although abundant evidence has shown the important roles of LDs in tumor development, the molecular mechanisms by which LDs directly regulate tumor development remain unknown. Intracellular crosstalk between LDs and protumor signaling pathways has been reported to drive tumorigenesis.^[^ ^17^, ^18^ ^]^ As cytoplasmic organelles, LDs can act as platforms for protein–protein interactions. The LD surface hosts a diverse array of structural and functional proteins, such as DGAT2, Rab18, perilipin, and CCT, which collectively maintain LD homeostasis and regulate LD interactions with other cellular structures.^[^ ^19^, ^20^, ^21^ ^]^ We previously reported that LDs can recruit the Notch inhibitor Numb and the E3 ubiquitin‐protein ligase MDM2 to the surface of LDs, facilitating MDM2‐mediated Numb degradation and in turn activating Notch signaling.^[^ ^22^ ^]^ A recent study also revealed that LDs mediate the degradation of non‐LD proteins via the ubiquitin‒proteasome system to control their quality and quantity.^[^ ^23^ ^]^

As MDM2 is also a well‐known regulator of intracellular p53, this observation led us to hypothesize that the tumor suppressor p53 might be recruited to LDs and subsequently degraded via MDM2. Moreover, the role of p53 in regulating lipid metabolism has received increasing attention in recent decades.^[^ ^24^, ^25^ ^]^ For example, p53 is known to reduce lipid synthesis and promote lipid degradation.^[^ ^26^ ^]^ Nevertheless, to date, no studies have investigated the impact of lipid or LD accumulation on p53 activity, stability, and function. Consequently, further investigations of the relationship between LDs and p53 are needed.

In this study, we aimed to determine whether and how LDs initiate the MDM2‐mediated p53 degradation in tumor cells. By analyzing clinical data and tumor organoid models, we revealed for the first time that p53 is located in triglyceride‐induced LDs and further clarified the mechanism by which LDs promote the MDM2‐mediated ubiquitination of p53 via the complex of p53 and Myh9, which is recruited to LDs by Cyb5r3. Moreover, we identified a positive regulatory loop involving LDs and p53 in tumor development that includes triglycerides, MDM2, p53, Myh9, Cyb5r3, RPS3A, C/EBPβ, PPARγ and CD36. Our findings reveal a direct role for LDs in tumor development and provide a novel mechanism by which MDM2‐mediated p53 degradation is initiated, resulting in treatment and dietary suggestions for cancer patients.

## 2 Results

### 2.1 Hypertriglyceridemia is Positively Associated with Tumor Progression

**Figure 1:**
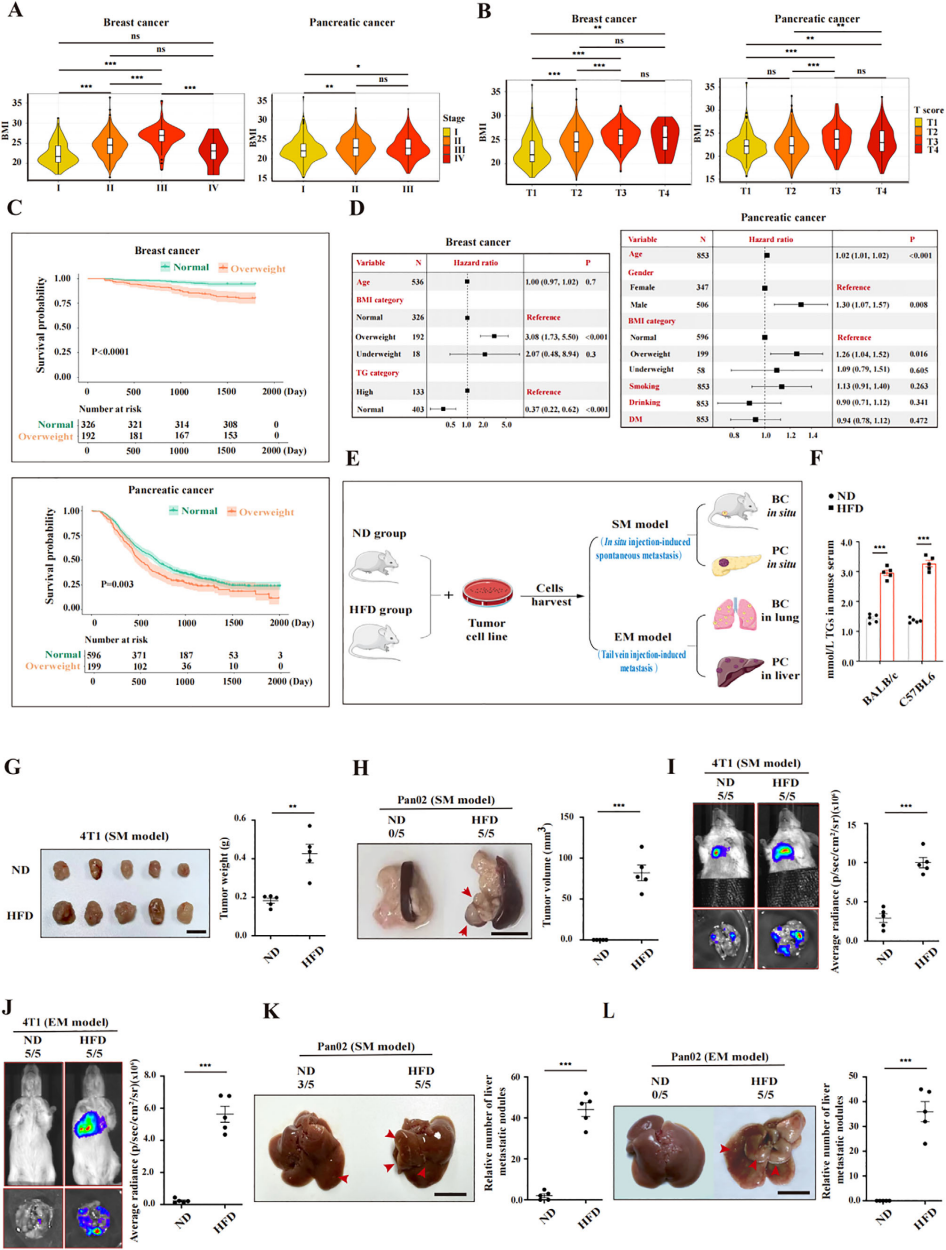
Hypertriglyceridemia is positively associated with tumor progression in patients with breast and pancreatic cancer. A) Correlation of BMI with TNM stage (AJCC, 8th ed.) in breast (*n* = 673) and pancreatic (*n *= 854) cancer, shown with violin plots. B) Correlation of BMI with T stage (AJCC, 8th ed.) in breast (*n* = 673) and pancreatic (*n* = 854) cancer, shown with violin plots. C) Kaplan–Meier survival curves comparing normal weight (green) and overweight (orange) individuals in the breast *(n* = 518) and pancreatic (*n* = 795) cancer groups. D) Multivariate Cox regression analysis of BMI and survival in the breast (*n* = 536) and pancreatic (*n* = 853) cancer cohorts. E) Schematic of mouse models for experimental and spontaneous metastasis. F) Serum triglyceride levels in mice on normal (ND) or high‐fat (HFD) diets, analyzed by one‐way ANOVA. G,H) Tumor images and weights from 4T1 (G) and Pan02 (H) xenografts in ND and HFD mice. I,J) Bioluminescence images and quantification of lung metastases in 4T1 SM (I) and EM (J) models. K,L) Liver metastases in the Pan02 SM (K) and EM (L) models. The red arrow indicates the metastasis. Statistical tests: Wilcoxon test for A and B; Student's *t‐*test for G‐L; one‐way ANOVA with post hoc test for F. Significance: ***, *p* < 0.001; **, *p* < 0.01; *, *p* < 0.05; ns, *p *> 0.05. Scale bar: 1 cm. Error bars denote the s.e.m. in F‐L (*n* = 5 per group). ND: normal diet; HFD: high‐fat diet; SM: spontaneous metastasis; EM: experimental metastasis; TG: triglycerides; BC: breast cancer; PC: pancreatic cancer.

To clarify the relationship between lipid accumulation and tumor development, we first analyzed the relationship between hyperlipidemia and tumor progression in patients. By analyzing the clinical lipid‐related parameters, such as the body mass index (BMI) and blood triglyceride levels, from 673 breast cancer patients and 854 pancreatic cancer patients, which are strongly associated with a high‐fat diet, we found that a high BMI was positively associated with more advanced tumor progression, including larger tumor size, metastases and lower survival rates (**Figure** **1**A–C). Through multivariate analysis, we further confirmed that overweight individuals with higher BMIs had poorer prognoses than those with normal BMIs (Figure 1D). Further analysis of the correlation between hypertriglyceridemia and tumor progression revealed that hypertriglyceridemia was a risk factor for tumor progression (Figure 1D). These findings provide additional evidence that hypertriglyceridemia is associated with accelerated disease progression and a shorter lifespan in tumor patients with tumors.

To confirm the protumor effect of hypertriglyceridemia, we established tumor models with or without a high‐fat diet (HFD). Briefly, the six‐week‐old female mice were randomly divided into two groups: the normal diet (ND) group, which was fed a 10% fat diet, and the HFD group, which was fed a 60% fat diet (Figure 1E). After eight weeks of continuous ND or HFD feeding, the serum triglyceride levels were significantly elevated in HFD‐fed mice (Figure 1F). Then, spontaneous metastasis (SM) and experimental metastasis (EM) tumor models were established in each group (Figure 1E). In the SM model, 4T1 breast cancer cells were injected into the mammary gland or Pan02 pancreatic cancer cells were injected into the pancreas. In the EM model, tumor cells were injected into the peripheral blood through the tail vein of mice. Once the tumor cells were inoculated, the tumor growth was monitored. We observed that the tumor volumes in situ derived from 4T1 or Pan02 cells were greater in the mice fed the HFD than in those fed the ND (Figure 1G,H). Importantly, the lung metastases of the 4T1 tumors (Figure 1I,J) and liver metastases of the Pan02 tumors were markedly greater in HFD‐fed mice than in those fed an ND, in both in the SM and EM metastasis models (Figure 1K,L). Collectively, these data indicate that lipid accumulation accelerates tumor growth in both in situ tumors and metastatic tumors.

### 2.2 Intracellular Lipid Drops Promote Tumor Growth

**Figure 2:**
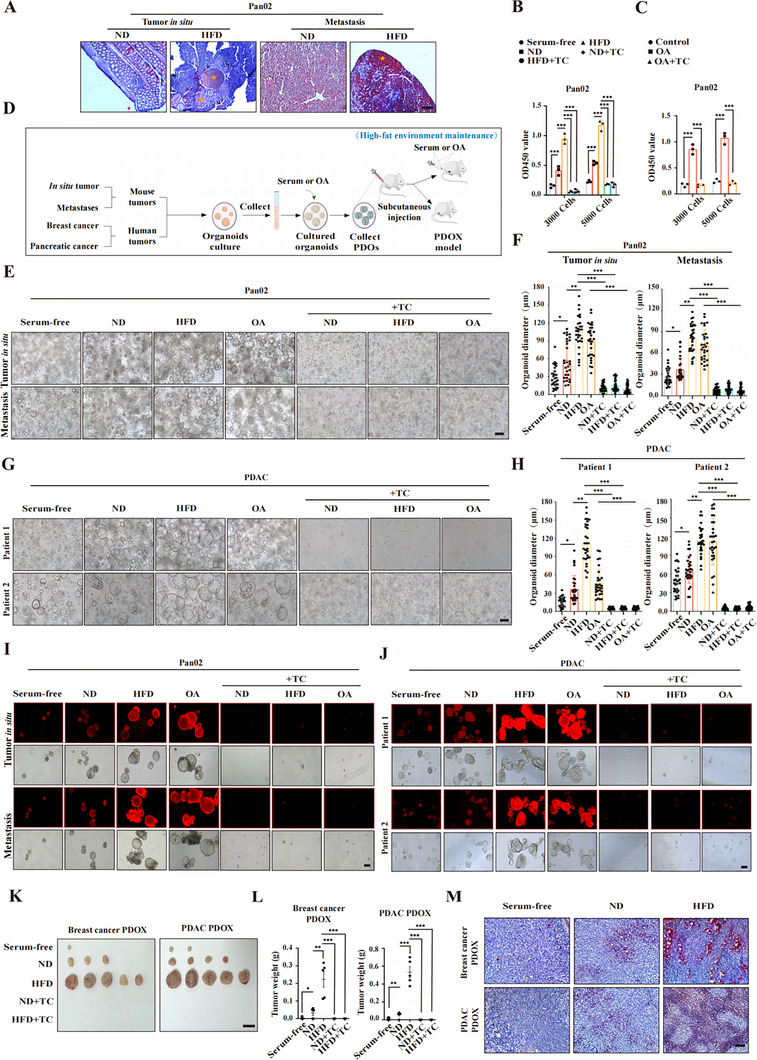
Intracellular lipid drops promote tumor growth. A) Oil Red O stains lipids (red) in tumor tissues (yellow stars). B) A CCK8 assay was used to measure cell proliferation in media with or without TC in 10% mouse serum, and the control group was serum‐free. C) A CCK‐8 assay was used to assess the effects of OA or TC effects on cell proliferation. D) Schematic of the experimental models used to test the ability of lipid accumulation to promote tumor growth. Breast and pancreatic cancer tissues from mice or patients were used to create organoids or PDOs, followed by PDOXs. High‐fat cell models were generated by serum or OA stimulation of tumor organoids. E,F) Pan02 organoids from pancreatic tumors and liver metastases were cultured in serum‐free or 10% serum media with or without OA or TC. E) Representative images and F) quantification of the organoid number; G‐H) PDAC organoids from patients cultured in serum‐free or 10% human serum media with or without OA or TC. G) Representative images and H) quantification of organoid number. I) Lipid accumulation in Pan02 organoids from pancreatic tumors and liver metastases cultured with or without mouse serum or OA or TC. J) Lipid accumulation in PDAC organoids from patients cultured with or without human serum or OA or TC. K,L) PDOX tumor development from breast cancer or PDAC organoids in 10% serum with or without TC in NOG mice. K) Images and L) tumor weights. M) Oil Red O staining of PDOX tumor tissues showing lipid accumulation (red). Experimental conditions: human ND serum from healthy BMI patients and human HFD serum from overweight patients; mouse ND serum from normal diet groups and mouse HFD serum from high‐fat diet groups; and 100 µm OA or 3 µm TC. Statistical analysis: Error bars represent the s.d. in B and C (*n *= 3 per group), s.e.m. in F, H (*n* = 30 per group), and I (*n* = 5 per group). One‐way ANOVA with post hoc tests for B, C, F, H, and L. Significance: ***, *p* < 0.001; **, *p* < 0.01; *, *p* < 0.05. Scale bars: 100 µm (A, E, G, I, J, and M); 1 cm (K). Control: fetal bovine serum; ND: normal diet; HFD: high‐fat diet; OA: oleic acid; TC: triacsin C; PDO: patient‐derived organoid; PDOX: patient‐derived organoid xenograft; PDAC: pancreatic ductal adenocarcinoma.

Previous studies have shown that free fatty acids formed by triglyceride metabolism can be absorbed by cells to form intracellular triglycerides, which are stored as LDs in cells.^[^ ^15^ ^]^ To clarify the mechanism by which lipid accumulation promotes tumor growth, LDs were observed in tumors from both the in situ sites and metastatic sites from HFD‐fed mice. As shown in **Figures** **2**A and S1A (Supporting Information), we detected many LDs in tumors from HFD‐fed mice, suggesting a close relationship between LDs and tumor development. To explore this, we first measured the proliferation of tumor cells cultured with conditional medium supplemented with serum from ND‐ or HFD‐fed mice. We observed that serum from HFD‐fed mice significantly promoted the formation of LDs in tumor cells (Figure S1B,C, Supporting Information), such as the mouse triple‐negative breast cancer (TNBC) cell line 4T1 and the pancreatic ductal adenocarcinoma (PDAC) cell line Pan02; moreover, the proliferation of tumor cells was markedly accelerated under the stimulation with an HFD (Figure 2B; Figure S1D, Supporting Information). In contrast, this pro‐proliferative ability was significantly inhibited by the addition of the lipogenesis inhibitor triacsin C (Figure 2B; Figure S1D, Supporting Information), suggesting that LD formation promotes tumor growth. These findings were reproduced in the human TNBC cell line MDA‐MB‐231 and the human PDAC cell line PANC1 (Figure S1B–D, Table S1, Supporting Information).

To confirm that lipid drops facilitate the proliferation of tumor cells, we stimulated mouse or human tumor cell lines with oleic acid (OA), a fatty acid that is abundant in the diet and is well known to induce intracellular lipid accumulation.^[^ ^27^ ^]^ Similar to the serum from HFD‐fed mice, OA alone significantly promoted the proliferation of tumor cell lines, and this effect was antagonized by triacsin C (Figure 2C; Figure S1E, Supporting Information). Therefore, these results confirm that lipid accumulation in tumor cells promotes tumor growth by the formation of lipid drops.

To further validate the protumor effect of intracellular lipid drops, we next investigated whether lipid accumulation promotes tumor organoid growth. By culturing tumor organoids with or without high‐triglyceride serum or OA in the presence or absence of triacsin C (Figure 2D), we observed that tumor organoids stimulated with high‐triglyceride serum or OA had larger organoid diameters than those in the other groups did, where the organoid diameter reflects the growth capacity of the organoids (Figure 2E–H; Figure S1F–L, Supporting Information). Moreover, the addition of triacsin C significantly inhibited lipid accumulation and organoid growth (Figure 2E–J; Figures S1F–L,S2A–F, Supporting Information). Similar results were obtained in human breast cancer or pancreatic cancer‐derived tumor organoids (Table S2, Supporting Information).

We obtained patient‐derived TNBC and PDAC tumor organoids and transplanted them into immunodeficient mice to establish patient‐derived organoid‐based xenografts (PDOXs). We observed that stimulation with high‐triglyceride serum or OA significantly increased the tumorigenicity and xenograft tumor growth capacity of the tumor organoids (Figure 2K,L; Figure S2G,H, Supporting Information). Subsequent analysis of lipid accumulation in PDOX tissues revealed that stimulation with high‐triglyceride serum or OA also significantly increased intratumoral lipid accumulation (Figure 2M; Figure S2I, Supporting Information). These findings show that a high‐lipid environment leads to increased intracellular lipid drops in tumor cells, resulting in enhanced tumorigenicity and growth.

### 2.3 Lipid Drops in Tumor Cells Promote p53 Degradation

**Figure 3:**
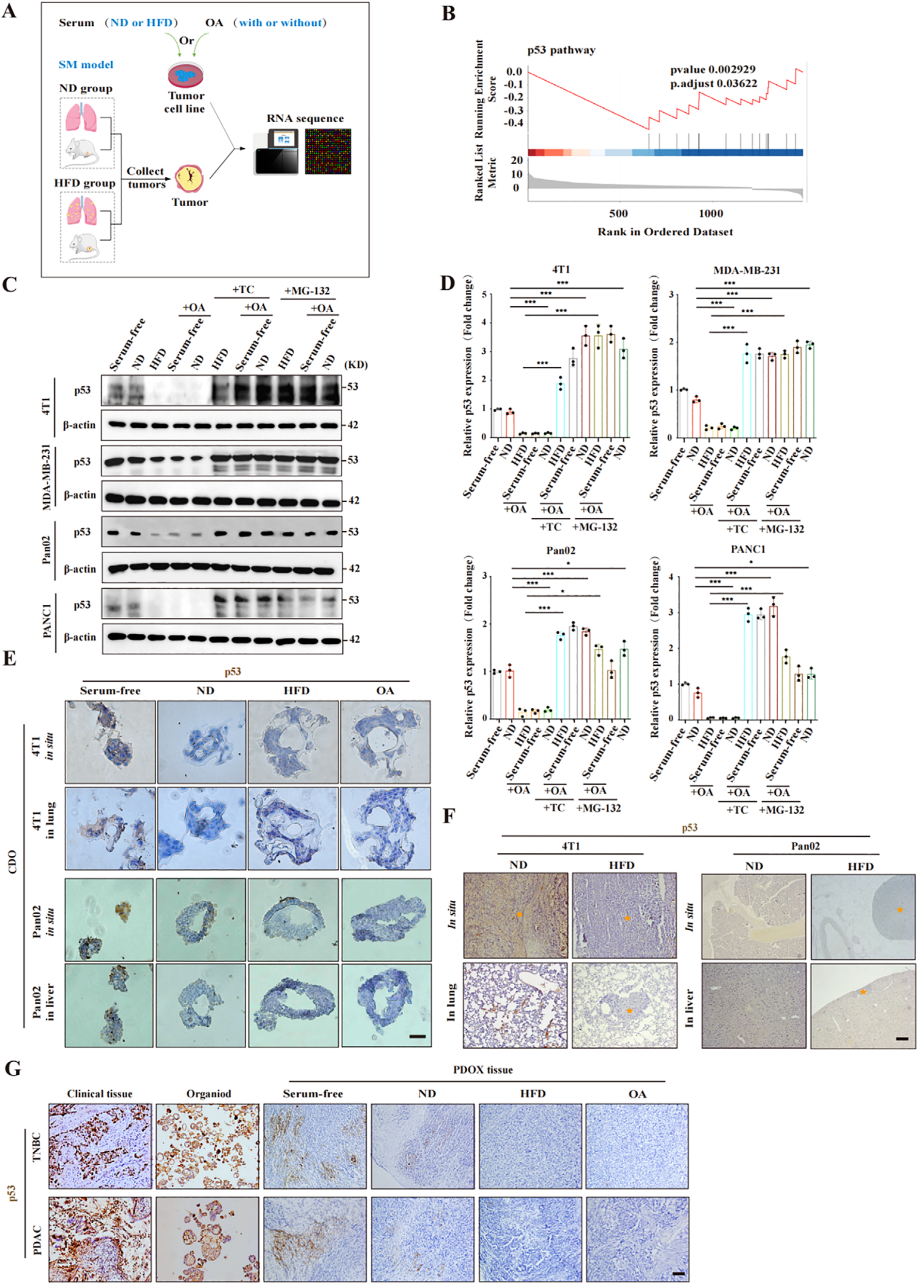
Lipid droplets in tumor cells promote p53 degradation. A) Schematic of experimental models used to explore how lipid accumulation enhances tumor growth at the cellular and whole‐organism levels. 4T1 cells were cultured with mouse serum and stimulated with OA to create a high‐fat model, followed by RNA sequencing. B) Gene set enrichment analysis of differentially expressed genes in 4T1 in situ tumors from the ND and HFD groups. The p53 pathway is highlighted. C,D) Western blot analysis of p53 expression in tumor cells treated with or without 10% mouse or human serum, OA, TC, or MG‐132. Fetal bovine serum was used as a control. β‐actin served as a loading control. C) Representative images and D) quantification. E) DAB staining of p53 accumulation in tumor organoids from in situ tumors and metastases cultured with or without mouse serum or OA. F) DAB staining of p53 accumulation in tumor tissues (yellow stars) from mice fed ND or HFD diets. G) DAB staining of p53 accumulation in clinical tissues, tumor organoids, and PDOX tissues from the same cancer patients (one TNBC and one PDAC). Tumor organoids were cultured under ND or HFD conditions with human serum. Experimental conditions: human ND serum from healthy BMI patients and human HFD serum from overweight patients; mouse ND serum from normal diet groups and mouse HFD serum from high‐fat diet groups; and 100 µm OA, 3 µm TC or 10 µm MG‐132. Scale bars: 10 µm (E), and 100 µm (F,G). Statistical analysis: One‐way ANOVA with post hoc test for D. Significance: ***, *p* < 0.001; *, *p* < 0.05. ND: normal diet; HFD: high‐fat diet; OA: oleic acid; TC: triacsin C; CDO: cell line‐derived organoid; PDOX: patient‐derived organoid xenograft; SM: spontaneous metastasis; TNBC: triple‐negative breast cancer; PDAC: pancreatic ductal adenocarcinoma.

To explore the molecular mechanisms underlying the protumor ability of intracellular lipid drops, we performed RNA sequencing analyses to compare the transcriptional profiles of 4T1 cells stimulated with high‐triglyceride serum or OA and of in situ or metastatic tumor tissues from mice fed the ND or HFD (**Figure** **3**A; Figure S3A, Supporting Information). In 4T1 cells, pathways associated with cell proliferation, such as the p53 pathway, JAK‐STAT pathway, and ribosome biogenesis pathway, were upregulated in the HFD serum‐stimulated group compared with those in the ND serum‐stimulated group (Figure S3B, Supporting Information). The upregulation of these pathways was even more striking in the OA‐stimulated group (Figure S3C–E, Supporting Information). Moreover, RNA‐seq data from in situ and metastatic tumor tissues revealed that HFD consumption upregulated not only pathways associated with cell proliferation but also pathways related to tumor metastasis, such as cell proliferation and cell adhesion pathways (Figure S3F–I, Supporting Information). These findings suggest that increased lipid accumulation plays an active role in tumor growth.

Further analysis of the RNA‐seq data from the normal and the high‐fat diet groups in the 4T1 in situ tumors revealed that lipid accumulation led to the downregulation of genes associated with the p53 pathway (Figure 3B) and the upregulation of genes associated with the MYC pathway (Figure S3J, Supporting Information). As a classical tumor suppressor protein, p53 plays a crucial inhibitory role in tumor occurrence and development by controlling processes such as the cell cycle, DNA repair, apoptosis, and senescence.^[^ ^28^ ^]^ Therefore, we hypothesized that lipid accumulation may promote tumor growth by facilitating the degradation of p53. In support of this hypothesis, stimulation with high‐triglyceride serum or OA decreased the level of p53 protein in tumor cells (Figure 3C,D), whereas treatment with triacsin C increased the level of p53 protein in tumor cells, although the level of p53 gene expression remained unchanged (Figure 3C; Figure S4A, Supporting Information), suggesting posttranscriptional modification of p53. Similar findings were observed in tumor organoids, tumor tissue from tumor‐bearing mice, and PDOX models (Figure 3E–G). Posttranscriptional modification of p53 was confirmed through the use of the proteasome inhibitor MG‐132, which led to an increase in the intracellular p53 protein level (Figure 3C). Taken together, these findings indicate that lipid accumulation promotes p53 ubiquitination and degradation.

### 2.4 Lipid Drops Enhance MDM2‐Mediated p53 Degradation by the Interaction of Cyb5r3 with Myh9

**Figure 4:**
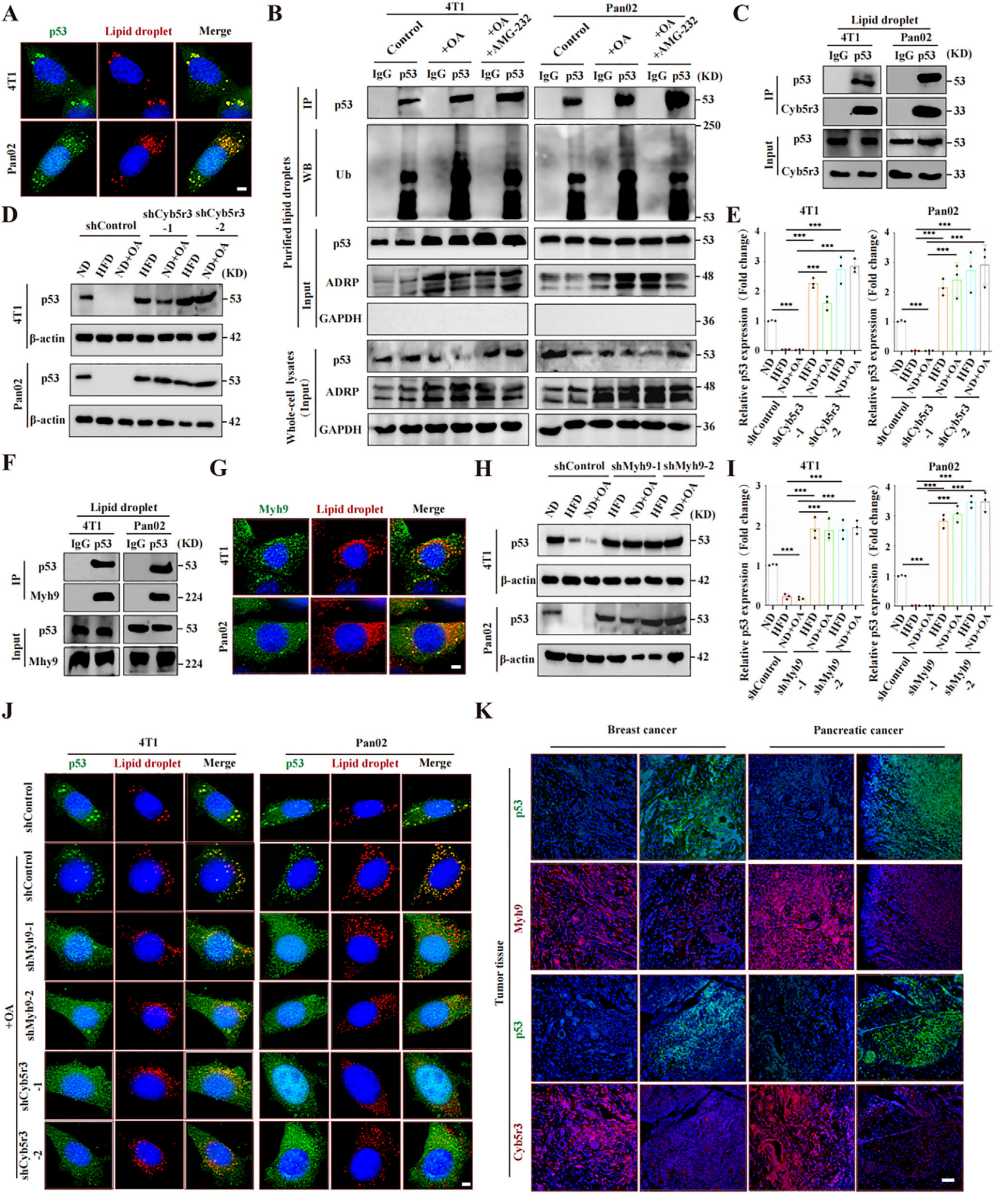
Lipid droplets enhance MDM2‐mediated p53 degradation via the Cyb5r3‐Myh9 interaction. A) Fluorescence imaging showing that anti‐p53 immunofluorescence (green) colocalized with lipid droplets (LDs) stained with Nile red (red). B) Immunoprecipitation and Western blot analysis of MDM2‐mediated p53 ubiquitination (Ub) on LDs. LDs were purified from equal numbers of control, OA‐treated, and OA+AMG‐232‐treated tumor cells. AMG‐232 inhibits MDM2 activity. ADRP and GAPDH label LDs and the cytosolic fraction, respectively. Fetal bovine serum was used as a control. C) Immunoprecipitation and Western blot analysis demonstrated the interaction between p53 and Cyb5r3 on purified LDs from tumor cells. D,E) Western blot analysis of p53 levels in tumor cells with Cyb5r3 knockdown (shCyb5r3‐1 and shCyb5r3‐2) cultured with mouse or human serum. D) Representative images and E) quantification. F) Immunoprecipitation and Western blot analysis of the interaction between p53 and Myh9 on purified LDs from tumor cells. G) Fluorescence imaging reveals Myh9 localization on LDs, visualized by Nile red staining. H,I) Western blot analysis of p53 levels in tumor cells with Myh9 knockdown (shMyh9‐1 and shMyh9‐2) cultured with mouse or human serum. H) Representative images and I) quantification. J) Fluorescence imaging shows the effect of Myh9 and Cyb5r3 knockdown on p53 localization to LDs in OA‐treated tumor cells. K) Immunofluorescence analysis of breast and pancreatic cancer tissues derived from obese patients (BMI ≥ 30 to validate the relationship between Myh9/Cyb5r3 and p53 expression. Experimental conditions: human ND serum from healthy BMI patients and human HFD serum from overweight patients; mouse ND serum from normal diet groups and mouse HFD serum from high‐fat diet groups; and 100 µm OA or 4 µm AMG‐232. β‐actin was used as a control for Western blotting. Scale bars: 5 µm (A,G,J) and 100 µm (K). Statistical analysis: One‐way ANOVA with post hoc test for E and I. Significance: ***, *p* < 0.001. Control: fetal bovine serum; ND: normal diet; HFD, high‐fat diet; OA: oleic acid.

Previous studies have shown that p53 undergoes ubiquitin‐dependent degradation. Moreover, we previously reported that MDM2, a well‐known E3 ubiquitin ligase, is located on LDs.^[^ ^22^ ^]^ We therefore speculated that the observed p53 degradation might be mediated by MDM2 on LDs. Subsequent Western blot analysis revealed that HFD reduced p53 phosphorylation levels, a condition conducive to ubiquitination‐mediated degradation of the protein. Interestingly, MDM2 phosphorylation remained unaffected under HFD treatment (Figure S4B,C, Supporting Information). Fluorescence imaging revealed that p53 indeed localized to LDs (**Figure** **4**A; Figure S4D, Supporting Information). To investigate whether MDM2 ubiquitinates p53 on LDs, we employed a well‐established method to isolate LDs from control cells and cells stimulated with OA.^[^ ^29^ ^]^ Adipose differentiation‐related protein (ADRP) and GAPDH were used as markers for LDs and the cellular membrane fraction, respectively. We then assessed the level of ubiquitinated p53 on LDs by immunoprecipitating p53 from control and OA‐stimulated cells and subjecting equal amounts of the precipitated sample to Western blotting with a ubiquitin antibody. We found that ubiquitinated p53 was indeed present in LDs (Figure 4B; Figure S4E, Supporting Information). Furthermore, the level of ubiquitinated p53 increased significantly as the number of LDs increased in OA‐stimulated cells (Figure 4B; Figure S4E, Supporting Information). Conversely, pretreatment of cells with the MDM2 inhibitor AMG‐232 led to a significant decrease in the level of ubiquitinated p53 (Figure 4B; Figure S4E, Supporting Information), indicating that the ubiquitination of p53 on LDs is mediated by MDM2. Our previous research revealed that UBX domain‐containing protein 8 (UBXD8) recruits the valosin‐containing protein (VCP)/MDM2 complex to LDs, where MDM2 ubiquitinates target proteins, and that VCP extracts ubiquitin‐tagged target proteins for degradation in the proteasome.^[^ ^22^ ^]^

To investigate how p53 is recruited to LDs, we purified LDs and performed immunoprecipitation of purified LD proteins with a p53 antibody, followed by mass spectrometry analysis which identified cytochrome b5 reductase 3 (Cyb5r3) and Myosin‐9 (Myh9) as LD p53 interactors (Figure S4F, Supporting Information). Immunoprecipitation confirmed that p53 and Cyb5r3 interact on purified LDs from tumor cells (Figure 4C; Figure S4G, Supporting Information), and Cyb5r3 is a robust LD protein.^[^ ^19^ ^]^ Knocking down Cyb5r3 significantly increased p53 protein levels (Figure 4D,E; Figure S4H–J, Supporting Information). Next, we used GenGLip 3 (http://cismu.net/genclip3/analysis.php) to construct the Myh9 interaction network, which revealed that Myh9 interacts with many molecules that play key roles in tumors, including p53 and MYC.^[^ ^30^ ^]^ Therefore, we hypothesized that Myh9 may recruit p53 to LDs by interacting with proteins on LD membranes. Immunoprecipitation also confirmed that p53 and Myh9 indeed interact on purified LDs from tumor cells (Figure 4F; Figure S5A, Supporting Information). Fluorescence imaging further revealed that Myh9 colocalized with LDs in OA‐stimulated cells (Figure 4G; Figure S5B, Supporting Information). Finally, shRNA‐mediated knockdown of Myh9 (Figure S5C, Supporting Information) increased p53 protein levels (Figure 4H,I; Figure S5D,E, Supporting Information) and suppressed the recruitment of p53 to LDs (Figure 4J; Figure S5F,G, Supporting Information), indicating that Myh9 facilitates p53 recruitment to LDs. These findings led us to speculate that Myh9 potentially interacts with Cyb5r3, which we further confirmed by Western blotting (Figure S5H, Supporting Information). Knocking down Cyb5r3 significantly suppressed p53 and Myh9 localization to LDs (Figure 4J; Figure S5F,G,I,J, Supporting Information), resulting in increased p53 protein levels (Figure 4D,E). Fluorescence imaging of tumor specimens from obese patients further revealed an inverse correlation between p53 protein levels and Myh9/Cyb5r3 expression (Figure 4K), reinforcing this molecular mechanism in human pathology. Taken together, these data suggest that Myh9 transports p53 to LDs as cargo through Cyb5r3.

### 2.5 p53 Degradation Promotes the Intracellular Accumulation of Lipid Drops through Regulating the Entry of RPS3A and C/EBPβ Complex into the Nucleus

**Figure 5:**
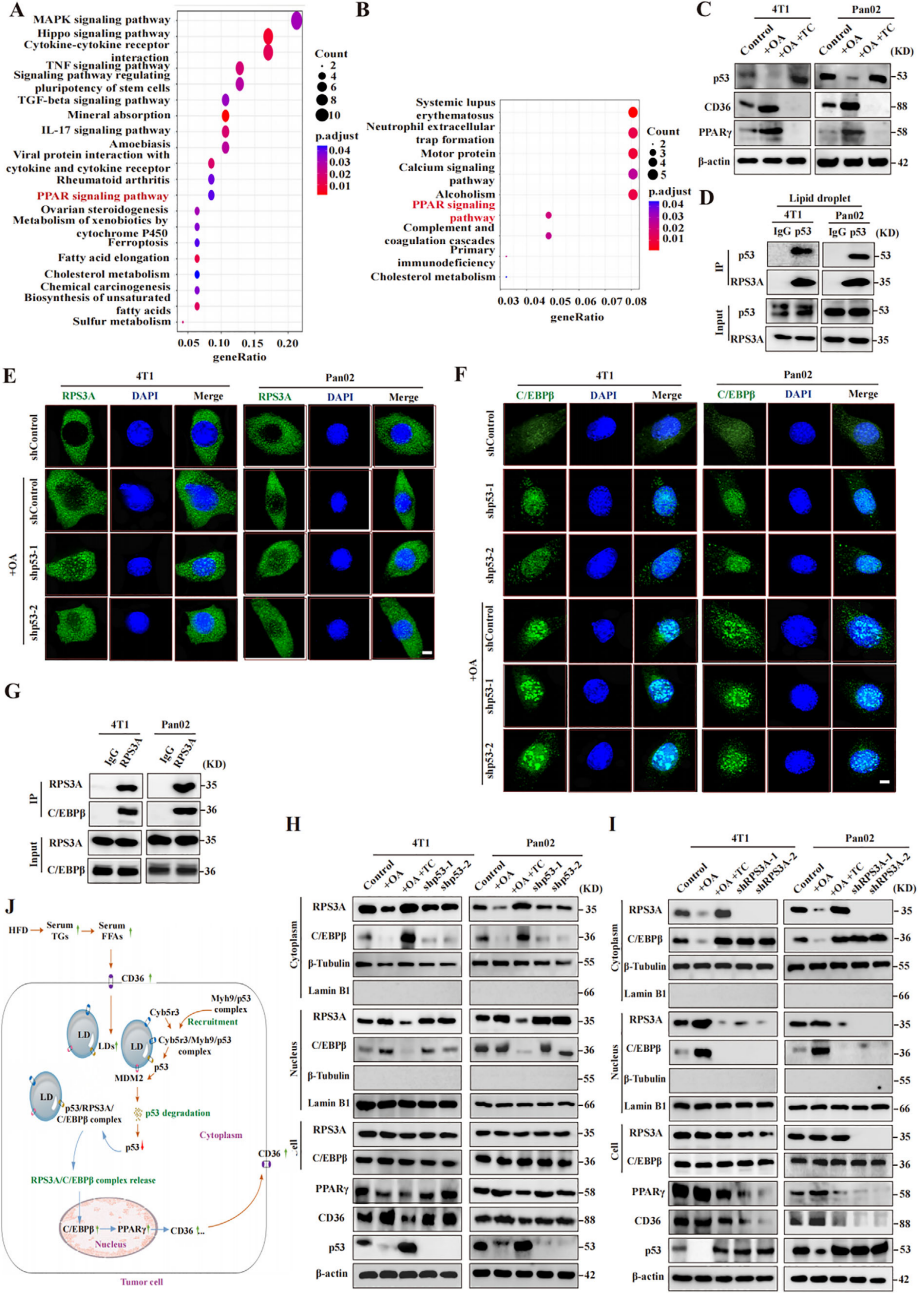
p53 degradation enhances intracellular lipid accumulation by regulating the nuclear import of the RPS3A‐C/EBPβ complex. A) Gene Ontology analysis of differentially expressed genes in 4T1 cells treated with ND or HFD serum, highlighting the role of the PPAR pathway in CD36 upregulation. B) Gene Ontology analysis of differentially expressed genes in 4T1 cells treated with or without oleic acid, emphasizing the involvement of the PPAR pathway in CD36 upregulation. C) Western blot analysis of p53, CD36, and PPARγ expression in tumor cells treated with OA and TC. D) Immunoprecipitation and Western blot demonstrating the interaction between p53 and RPS3A on purified LDs from tumor cells. E,F) Fluorescence imaging staining showing the effect of p53 knockdown (shp53‐1 and shp53‐2) on the nuclear localization of RPS3A (E) and C/EBPβ (F) in OA‐treated tumor cells, G) Immunoprecipitation and Western blot analysis showing the interaction between RPS3A and C/EBPβ in tumor cells, H,I) Western blotting of nuclear and cytoplasmic fractions showing the effects of p53 knockdown (shp53‐1 and shp53‐2) (H) and RPS3A knockdown (shRPS3A‐1 and shRPS3A‐2) (I) on the cytoplasmic or nuclear localization of RPS3A and C/EBPβ in tumor cells treated with OA and TC. J) Schematic model proposing that p53 degradation leads to CD36 upregulation, allowing increased lipid droplet accumulation in tumor cells and further promoting p53 degradation, forming a positive feedback loop. Experimental conditions: tumor cells were treated with 100 µm OA or 3 µm TC. β‐actin was used as a control for Western blotting. Scale bars: 5 µm (E,F). Control: fetal bovine serum; ND: normal diet; HFD: high‐fat diet; OA: oleic acid; TC: triacsin C.

The RNA‐seq analyses revealed a significant enrichment of the peroxisome proliferator‐activated receptor (PPAR) pathway in OA‐stimulated cells (**Figure** **5**A,B). A previous report revealed that the PPAR pathway plays a key role in the expression of CD36, an important fatty acid transporter,^[^ ^31^ ^]^ which facilitates the formation of lipid drops. Western blotting of control and OA‐stimulated tumor cells revealed that high‐fat‐induced p53 degradation is accompanied by the upregulation of PPARγ and CD36 expression (Figure 5C; Figure S6A,B, Supporting Information), suggesting that p53 degradation might upregulate CD36 expression, ultimately leading to the formation of lipid drops.

To explore the mechanism by which p53 degradation may regulate CD36 expression, we focused on the transcription factor C/EBPβ, which has been reported to act as a transcriptional activator of the PPARγ gene.^[^ ^32^ ^]^ We performed immunoprecipitation using an anti‐p53 antibody and identified RPS3A by mass spectrometry analysis (Figure S4F, Supporting Information). RPS3A has been reported to promote PPARγ expression^[^ ^33^ ^]^ and the transport of transcription factors such as NF‐κB from the cytoplasm to the nucleus.^[^ ^34^ ^]^ Immunoprecipitation analysis confirmed that p53 and RPS3A indeed interact on LDs purified from tumor cells (Figure 5D; Figure S6C, Supporting Information). Notably, fluorescence imaging further demonstrated the co‐localization of p53 and RPS3A on LDs (Figure S6D, Supporting Information). This spatial association led us to speculate that p53 on the LDs may bind to RPS3A and restrict its entry into the nucleus and that the ubiquitination and degradation of p53 on LDs may release this restriction. To test this hypothesis, we used two independent shRNAs to knock down p53 (Figure S6E, Supporting Information). Using fluorescence imaging, we observed that p53 knockdown promoted the accumulation of RPS3A in the nucleus (Figure 5E; Figure S6F,G, Supporting Information). Therefore, p53 inhibits the aggregation of RPS3A in the nucleus.

We next confirmed that C/EBPβ is involved in the process by which p53 regulates CD36 expression. First, we performed fluorescence imaging, which revealed that p53 knockdown promoted C/EBPβ localization to the nucleus (Figure 5F; Figure S7A,B, Supporting Information), which was consistent with the localization of RPS3A in the nucleus. Immunoprecipitation experiments confirmed that RPS3A and C/EBPβ interacted within tumor cells (Figure 5G; Figure S7C, Supporting Information). Western blotting also confirmed that p53 knockdown increased RPS3A and C/EBPβ accumulation in the nucleus, which in turn upregulated PPARγ and CD36 expression (Figure 5H; Figure S7D, Supporting Information). Second, we used two independent shRNAs to knock down RPS3A (Figure S7E, Supporting Information). Fluorescence imaging further revealed that RPS3A knockdown inhibited some C/EBPβ aggregation in the nucleus (Figure S7F,G, Supporting Information). Western blot analysis confirmed that RPS3A degradation reduced C/EBPβ accumulation in the nucleus, downregulated PPARγ, and CD36 expression, and upregulated p53 expression (Figure 5I; Figure S7H, Supporting Information). In addition, RPS3A knockdown inhibited the accumulation of LDs in tumor cells (Figure S7I,J, Supporting Information). Together, these data show that p53 binds to RPS3A in the cytoplasm, and when p53 is degraded, RPS3A binds C/EBPβ and transports it from the cytoplasm to the nucleus. This leads to the increased accumulation of C/EBPβ in the nucleus, which in turn upregulates PPARγ expression and ultimately leads to upregulation of CD36 expression. Consequently, more LDs accumulate in tumor cells and further p53 degradation is promoted, forming a positive feedback loop (Figure 5J).

### 2.6 Lipid Drops and the p53 Regulatory Circuit Promote Tumor Growth

**Figure 6:**
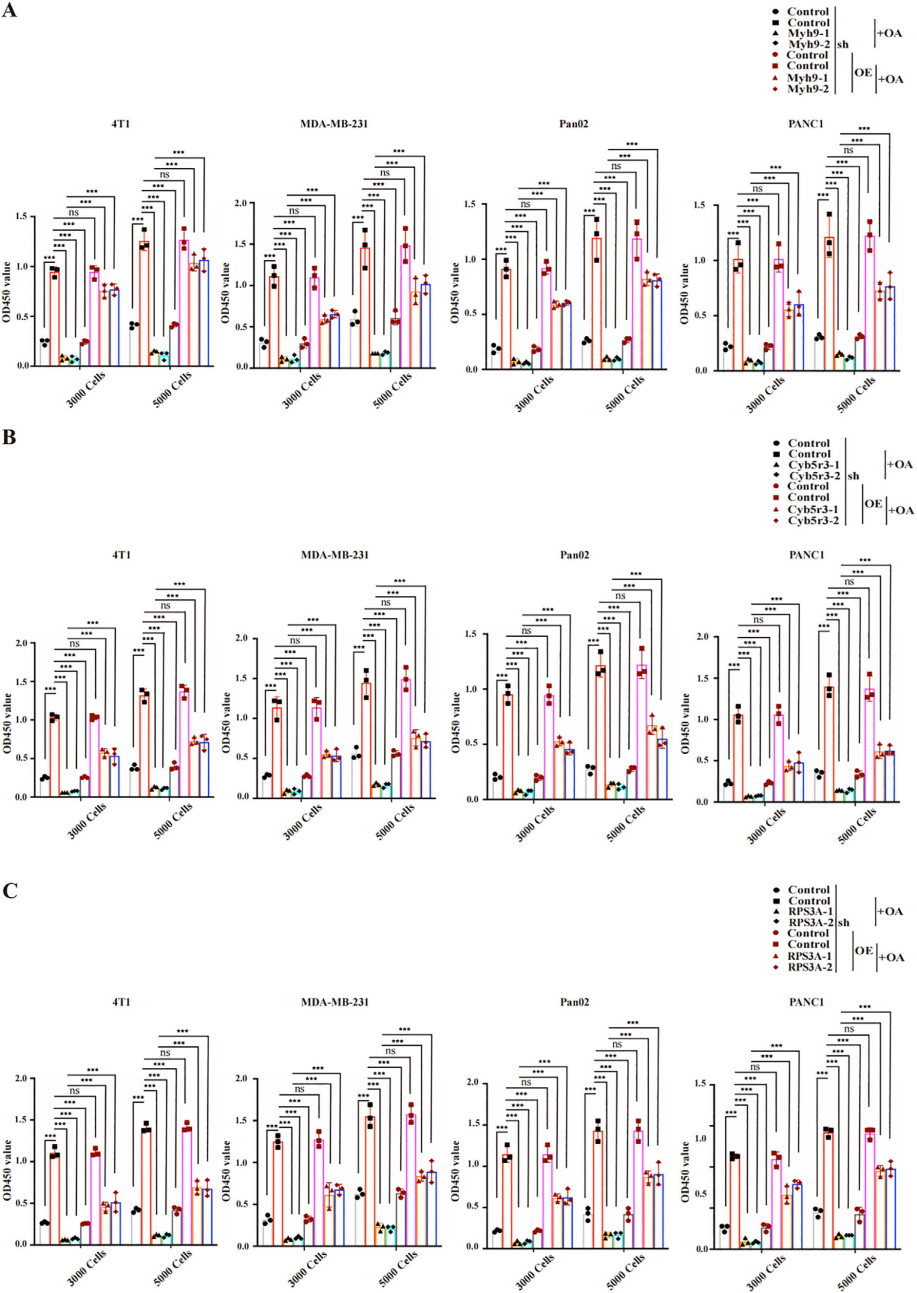
The lipid droplet‐p53 regulatory circuit enhances tumor growth. A) A CCK‐8 assay was used to measure the proliferation of Myh9‐knockdown (shMyh9‐1 and shMyh9‐2) tumor cells in response to 100 µm OA stimulation; B) A CCK‐8 assay was used to quantify the proliferation of Cyb5r3‐knockdown (shCyb5r3‐1 and shCyb5r3‐2) tumor cells upon 100 µm OA stimulation; C) A CCK‐8 assay was used to evaluate the proliferation of RPS3A‐knockdown (shRPS3A‐1 and shRPS3A‐2) tumor cells following 100 µm OA stimulation. Overexpression (OE) was used for the shRNA rescue experiments. The control conditions utilized fetal bovine serum, and OA denotes oleic acid treatment. Error bars denote the s.d. in A‐C (*n* = 3 per group). *P*‐values were calculated using one‐way ANOVA with a post hoc test in A, B, and C. ***, *p* < 0.001; ns, *p *> 0.05.

To clarify the critical role of the positive feedback loop of p53 and lipid drops in tumor development, we performed a knockdown of Myh9, Cyb5r3, or RPS3A in OA‐stimulated tumor cells and assessed proliferative capacity. Strikingly, silencing any of these three genes robustly inhibited tumor cell proliferation (**Figure** **6**). Importantly, this suppressive effect proved reversible‐overexpression of the corresponding gene in knockdown cells partially restored proliferative capacity (Figure 6; Figures S4H,s5C,S7E, Supporting Information). In addition, the knockdown of Myh9, Cyb5r3, or RPS3A inhibited tumorigenicity and tumor growth in a subcutaneous xenograft tumor model under HFD conditions (Figure S8A–F, Supporting Information). Moreover, we detected lipid accumulation and p53 expression in the aforementioned tumor tissue sections, confirming the roles of Myh9, Cyb5r3, and RPS3A in regulating p53 degradation through lipid accumulation at the tissue level (Figure S8G,H, Supporting Information). Therefore, the involvement of Myh9, Cyb5r3, and RPS3A mediate dynamic changes in lipid accumulation and p53 stability within tumors, thus affecting tumor growth. Together, these data indicate that LD‐mediated p53 degradation and p53 degradation‐induced LD formation promote tumor development, highlighting a new strategy for tumor therapy based on the LD‐p53 circuit.

### 2.7 Lipid Restriction Inhibits Tumor Growth

**Figure 7:**
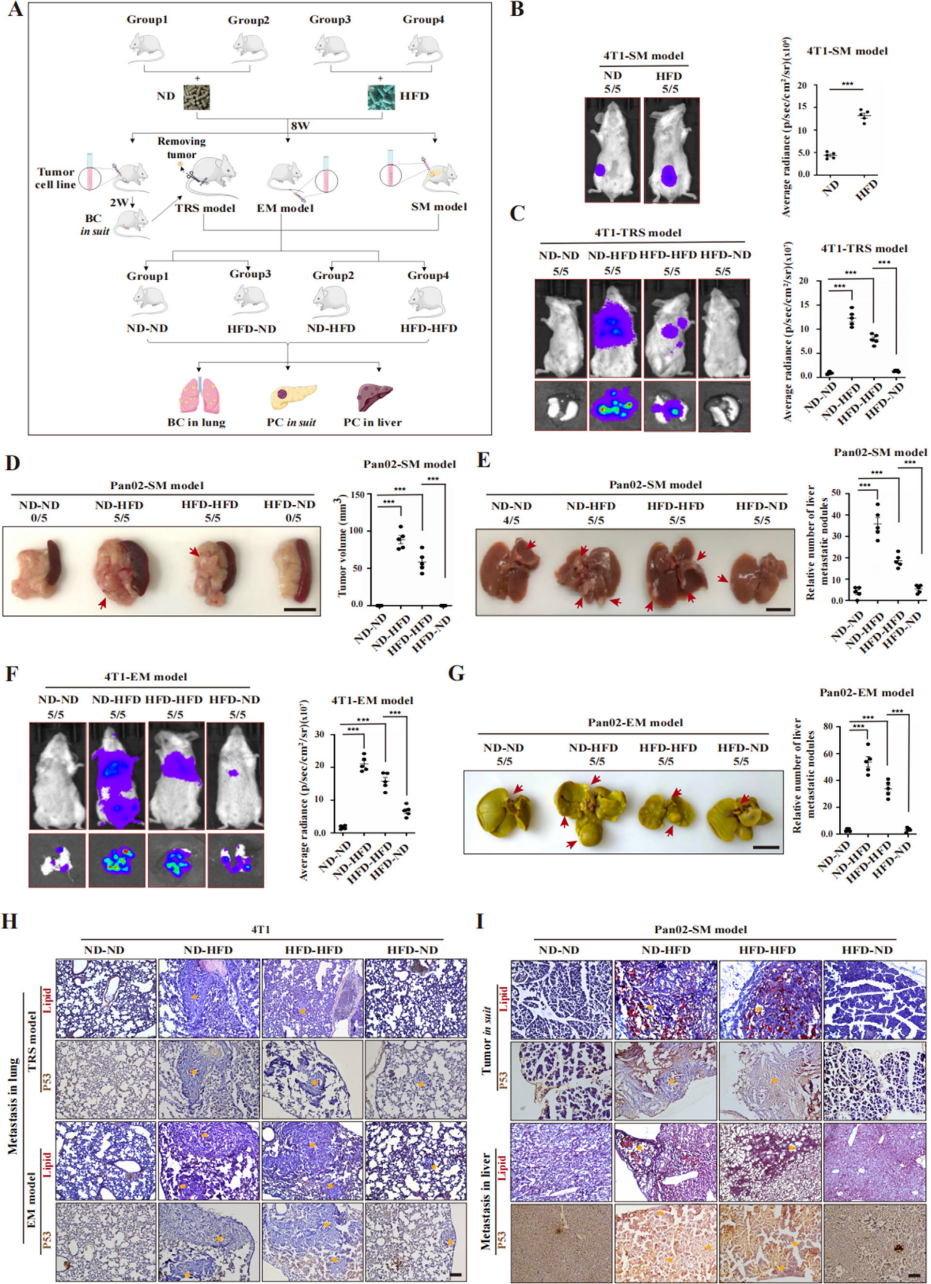
Lipid restriction impedes tumor growth. A) Experimental design illustrating the impact of dietary fat alterations on tumor progression in animal models. B,C) Bioluminescence imaging (left) and signal quantification (right) of 4T1 SM model tumors in vivo (B) and lung metastases ex vivo under TRS model conditions (C). D) Pan02 SM model: Photographs (left) and volume measurements (right) of xenograft tumors in response to dietary changes; E) Pan02 SM model: photographs (left) and quantification (right) of liver metastases following dietary changes; F) 4T1 EM model: bioluminescence images (left) and signal quantification (right) of lung metastases in vivo (upper) and *ex vivo* (lower) with dietary changes; G) Pan02 EM model: photographs (left) and quantification (right) of liver metastases under dietary changes. H,I) Oil Red O and DAB staining of tumor sections (yellow stars) showing lipid (red) and p53 accumulation in response to different diets. Red arrows indicate tumors or metastases. Error bars denote the s.e.m. in B‐G (*n* = 5 per group). Statistical analysis: Student's *t*‐test for B and one‐way ANOVA with post hoc test for C‐G. Significance levels: ***, *p *< 0.001. Scale bars: 100 µm (H,I) and 1 cm (D, E and G). ND: normal diet; HFD: high‐fat diet; SM: spontaneous metastasis; EM: experimental metastasis; TRS: tumor resection and suture; BC: breast cancer; PC: pancreatic cancer.

On the basis of these findings, we speculated that reducing hypertriglyceridemia may be an effective approach for inhibiting tumor growth and extending survival. To validate this, we established a diet‐switching model in which we modified the dietary structure of tumor‐bearing mice to reduce their blood triglyceride levels and then examined the effect on subsequent tumor growth (**Figure** **7**A). We found that triglyceride levels were increased in the mice that switched from the ND to the HFD and decreased in the mice that switched from the HFD to the ND (Figure S9A,B, Supporting Information). Compared with the mice fed a sustained HFD, the mice in the sustained ND group presented significantly smaller tumors and extended survival (Figure S9C–F, Supporting Information). Importantly, tumor‐bearing mice that were switched from the ND to the HFD had faster tumor growth and shorter survival times than those on a maintained ND or HFD (Figure 7C–G). Interestingly, the mice that switched from the HFD to the ND exhibited slower tumor growth at both the primary and metastatic sites than did the mice that were maintained on the HFD or switched from the ND to the HFD (Figure 7B–G). These data suggest that reducing lipid intake helps inhibit tumor growth.

The results of histological staining for lipid accumulation and p53 expression in primary and metastatic tumor tissues from the diet‐switching model mice (Figure 7H,I; Figure S9G,H, Supporting Information) provided additional evidence supporting these findings. These results further confirmed that increased lipid accumulation facilitates p53 degradation. Collectively, these observations imply that altering dietary habits to decrease lipid intake and effectively manage and lowering blood triglyceride levels subsequent to tumor onset could improve patient prognosis and prolong survival, potentially benefiting cancer patients as well.

## 3 Discussion

Our previous research confirmed that MDM2 is recruited to LDs through the AAA+ ATPase VCP and the LD protein UBXD8.^[^ ^22^ ^]^ Thus, we hypothesize that following ubiquitination by MDM2 on lipid droplets (LDs), p53 may subsequently be extracted by the VCP for degradation within the proteasome. Additionally, our findings reveal that the transporter protein Myh9 and the LD‐associated protein Cyb5r3 facilitate the recruitment of p53 to LDs, which in turn promotes p53 ubiquitination by MDM2 at these sites. Furthermore, we have discovered that p53 plays a role in regulating LD accumulation. Specifically, we found that p53 inhibits the expression of the fatty acid transporter protein CD36, primarily by restricting the cellular localization of the ribosomal protein RPS3A. This restriction leads to alterations in the nuclear accumulation of C/EBPβ, ultimately affecting CD36 expression and thereby modulating LD accumulation within cells. These results contribute to a better understanding of the p53‐LD feedback loop and align with current knowledge regarding p53's role in fatty acid transport.

Previous work suggests that providing sufficient energy and nutrients, including the intake of proteins, energy, vitamins, and minerals, is important for supporting the immune system of cancer patients, improving their quality of life and treatment outcomes.^[^ ^35^ ^]^ It is worth noting that oleic acid, the fatty acid used in our research, is considered to have beneficial functions such as lowering cholesterol, protecting cardiovascular health, reducing oxidative damage, and regulating inflammation^[^ ^36^ ^]^ and may be considered a “healthy fat”. Nevertheless, oleic acid has been extensively used in scientific research for the construction of cellular lipid accumulation models.^[^ ^22^, ^27^ ^]^

Although prior studies have established a correlation between an HFD and tumor progression through mechanisms such as increased fatty acid synthesis, chronic inflammation, and gut microbiota dysregulation,^[^ ^37^, ^38^, ^39^ ^]^ our work reveals a previously unrecognized axis in which LDs directly orchestrate tumor suppressor inactivation. Unlike earlier models that broadly attributed the protumor effects of an HFD to systemic metabolic dysregulation,^[^ ^40^ ^]^ we demonstrated that LD biogenesis initiates a spatially organized proteolytic cascade, positioning these organelles as central regulators of oncogenic proteostasis. This mechanistic divergence is exemplified by our finding that HFD‐induced LDs recruit p53 via Cyb5r3‐Myh9 interactions, enabling localized ubiquitination and degradation of p53, which is a process distinct from canonical nucleocytoplasmic p53 regulation.^[^ ^41^ ^]^ Therefore, this study reconceptualizes the role of LDs in HFD‐driven tumorigenesis.

Given its potent tumor suppressive properties and the reliance of many established tumors on its inactivation, p53 stands out as a highly promising target for the development of novel anticancer drugs. However, as a transcription factor, p53 has traditionally been deemed undruggable. Despite this, several innovative strategies have been explored to target dysfunctional p53 in cancer treatment. For tumors expressing mutant p53, the primary strategy involves restoring tumor suppressor function using compounds that act generically or selectively target one or a few specific p53 mutations. Additionally, approaches to deplete mutant p53 or target vulnerabilities created by its expression are currently in development. In contrast, for tumors with wild‐type p53, the main approach focuses on protecting p53 from the inhibitory actions of MDM2 and MDMX by targeting these negative regulators with inhibitors. While the results of some clinical trials involving MDM2 inhibitors and compounds that restore mutant p53 function are encouraging, none of these agents have yet received FDA approval. As such, alternative strategies are gaining traction, driven by a deeper understanding of p53 biology, the mechanisms of action of compounds and treatment regimens, and the advent of new technologies, such as proteolysis‐targeting chimeras for MDM2 degradation.^[^ ^3^, ^10^ ^]^ In this study, we provided a lipid‐restrict strategy for maintaining the p53 stability.

A key advancement of our study lies in resolving the paradox of cytoplasmic p53 accumulation correlating with poor prognosis in patients with PDAC.^[^ ^42^ ^]^ Although earlier work interpreted cytoplasmic p53 as a dysfunctional pool,^[^ ^26^ ^]^ imaging revealed that much cytoplasmic p53 colocalizes with LDs, where it undergoes MDM2‐mediated degradation rather than exerting tumor‐suppressive functions. This LD‐specific degradation mechanism explains why cytoplasmic p53 fails to activate apoptotic pathways despite wild‐type TP53 status. Compared with previous reports linking an HFD to general p53 downregulation,^[^ ^43^ ^]^ our data provide spatial resolution, showing that LD membranes enhance the ubiquitination efficiency of MDM2 through conformational activation and substrate confinement, which is a phenomenon analogous to immune synapse signaling.^[^ ^44^ ^]^ These findings demonstrate spatial specificity: LDs serve as catalytic platforms for tumor suppressor degradation.

Our discovery of the RPS3A‐C/EBPβ nuclear‐shuttling mechanism extends the current understanding of metabolic reprogramming in HFD‐associated tumors. Although the Warburg effect emphasizes glycolysis,^[^ ^45^ ^]^ we identified a lipid‐centric circuit in which p53 degradation triggers PPARγ/CD36 upregulation, resulting in self‐reinforcing lipid addiction. This loop operates independently of classical p53 targets such as p21 and Bax,^[^ ^46^ ^]^ suggesting that LDs recruit a specialized p53 subpopulation dedicated to lipid metabolism regulation.

Current strategies targeting MDM2‐p53 interactions, exemplified by small‐molecule inhibitors such as AMG‐232, are limited by compensatory MDMX upregulation.^[^ ^9^ ^]^ Our study introduces two transformative approaches: 1) LD‐directed therapy, where triacsin C achieved tumor growth reduction in PDOX models with efficacy comparable to that of cisplatin while circumventing hematopoietic toxicity,^[^ ^47^ ^]^ which is a key advantage over conventional chemotherapeutics; and 2) metabolic adjuvants, as dietary reversion from an HFD to normal nutrition post‐tumor detection restored p53 levels, validating nutritional interventions as practical complements to MDM2 inhibitors. These mechanisms diverge from those of previous HFD studies emphasizing caloric restriction,^[^ ^48^ ^]^ instead prioritizing precise modulation of lipid uptake pathways.

Our research conducted a diet transition experiment on tumor mice by simulating the common dietary changes that cancer patients undergo after diagnosis or surgery. These changes usually include a large intake of nutrients, especially high‐fat foods, aimed at enhancing anti‐tumor immunity. However, our key findings suggest that the sudden adoption of a high‐fat diet is even more harmful than maintaining a high‐fat diet continuously. More importantly, reducing fat intake in the diet in a timely manner can help slow down the progression of the disease. These findings underscore the need for caution in cancer patients consuming fatty acids that may lead to cell lipid accumulation. In order to optimize the dietary management of cancer patients, we propose the following suggestions: First, avoid sudden changes in dietary habits, especially increasing the intake of high‐fat foods; Second, regularly monitor lipid levels and related biomarkers to evaluate the effectiveness of dietary adjustments; Finally, work closely with the medical team to develop a personalized diet plan. In addition, our research also points out the direction of future research, including exploring the impact of different types of fat on cancer progression and developing more effective dietary intervention strategies. We expect that these findings will provide valuable guidance for the dietary management of cancer patients.

Our study establishes that lipid droplet (LD) biogenesis in cancer cells drives tumor progression via MDM2‐mediated p53 degradation, unveiling a targetable metabolic vulnerability. While tumor LDs function as oncogenic signaling hubs, their roles in normal cells under lipid stress remain enigmatic. Notably, HFD‐induced LD accumulation occurs in both malignant and non‐tumor hepatocytes, reflecting a systemic adaptation to lipid overload.^[^ ^49^, ^50^ ^]^ In normal hepatocytes, transient LD formation acts as a cytoprotective mechanism, sequestering excess fatty acids to mitigate lipotoxicity and organelle stress—a hallmark of early‐stage NAFLD.^[^ ^51^ ^]^ Paradoxically, chronic LD retention in these cells correlates with elevated HCC risk in long‐term HFD models, revealing a context‐dependent duality: LDs transition from metabolic safeguards to precancerous liabilities.^[^ ^52^ ^]^

This duality prompts a critical question: Do malignant and normal cells exploit distinct LD‐protein networks to execute opposing functions? Our work identifies Cyb5r3/Myh9‐p53 as a tumor‐specific LD complex driving oncogenesis, yet analogous mechanisms in non‐transformed cells remain unexplored. Comparative LD proteomics across HFD‐exposed hepatocytes and tumors could reveal malignancy‐specific dependencies, guiding therapies (e.g., lipogenesis inhibitors) to disrupt oncogenic LD pathways while preserving physiological roles in energy storage and membrane synthesis. Deciphering this context‐dependent rewiring of LD biology may reconcile their dual roles in health and disease, enabling strategies to harness lipid metabolic plasticity without disrupting systemic homeostasis.

HFD could modulate p53 degradation through both ubiquitination and phosphorylation. Studies indicate that phosphorylation of p53 at serine 15 prevents its binding to MDM2, thereby suppressing ubiquitination‐dependent degradation and significantly extending p53's half‐life.^[^ ^53^ ^]^ The question of how HFD influences p53 phosphorylation is particularly intriguing and will be further explored in subsequent studies.

Our work represents a natural extension of our previous findings, in which we established the significance of lipid droplets and lipid metabolism in pancreatic ductal adenocarcinoma and colorectal cancer, respectively.^[^ ^22^, ^54^ ^]^ Overall, this research offers a novel perspective on how a high‐fat diet fuels tumor growth, provides fresh insights into the inhibition of tumor growth via the suppression of excessive lipid droplet (LD) formation, and provides a more precise scientific rationale for encouraging cancer patients to avoid a high‐fat diet.

## 4 Conclusion

This study reveals a novel pathway in which lipid droplets initiate MDM2‐mediated p53 degradation, which plays a pivotal role in tumorigenesis and progression. Our research elucidates a previously unknown mechanism by which the enrichment of lipid droplets and the Cyb5r3 molecule anchor the p53/Myh9 complex to these droplets, thereby facilitating MDM2 binding and accelerating p53 degradation within tumor cells. Furthermore, we discovered that the degradation of p53 triggers the release of the RPS3A‐C/EBPβ complex, which in turn promotes the expression of molecules associated with lipid droplet formation. Notably, dietary changes aimed at reducing lipid droplets in tumor cells significantly inhibit tumor growth and increase p53 levels. These findings not only provide a theoretical foundation for developing tumor treatment strategies that target the MDM2‐p53 pathway but also offer fresh insights into the intricate relationship between high‐fat diets and the development of tumors. In summary, our study reveals the mechanism through which lipid droplets regulate p53 degradation, highlighting the role of high‐fat diets in tumor progression and aiding in the development of therapeutic drugs targeting p53 instability.

## 5 Experimental Section

### Cell Culture

HEK293T cells, the murine mammary carcinoma 4T1 (luciferase‐expressing) cell line, the murine pancreatic ductal adenocarcinoma Pan02 cell line, the human breast cancer MDA‐MB‐231 cell line, and the human pancreatic cancer PANC1 cell line were obtained from the American Type Culture Collection (ATCC). 4T1 cells were cultured in RPMI‐1640 medium (GIBCO) supplemented with 10% FBS (PAN‐Biotech) and 1% penicillin‒streptomycin (HyClone). HEK293T, Pan02, MDA‐MB‐231, and PANC1 cells were cultured in DMEM (GIBCO) supplemented with 10% FBS and 1% penicillin‒streptomycin. In certain experiments, human ND serum was collected from healthy BMI patients and human HFD serum from overweight patients; mouse ND serum was also collected from normal diet groups and mouse HFD serum from high‐fat diet groups; these serum samples were used to culture tumor cells from the corresponding species, where 10% FBS was replaced with an equivalent volume of mouse or human serum. In some experiments, oleic acid (OA, 100 µm) was added to the tumor cell cultures to promote lipid accumulation. Conversely, in some experiments, triacsin C (TC, 3 µm) was added to the tumor cell cultures to inhibit lipid accumulation. All the cell lines were maintained at 37 °C in humidified 5% CO_2_ conditions and were routinely tested for mycoplasma contamination by PCR.

### Mice and Xenograft Formation

Six‐week‐old female BALB/c mice, C57BL/6 mice, and NOG mice were purchased from the Weitonglihua Corporation (Beijing, China) and used in the studies. The mice were maintained in a specific pathogen‐free facility with a 12 h:12 h light: dark cycle at 21–23 °C, routinely checked by certified veterinarians, and deemed healthy prior to the tumor‐bearing experiments. After tumor cell transplantation, the mice were monitored daily for any signs of suffering or abnormal behavior. Mouse maintenance and procedures were approved by the Biomedical Research Ethics Committee of the Institute of Biophysics, Chinese Academy of Sciences. The experimental procedures were performed following the relevant ethical regulations regarding animal research.

Both BALB/c and C57BL/6 mice were used for xenograft experiments. 4T1 cells and Pan02 cells were transplanted into BALB/c and C57BL/6 mice, respectively. In the spontaneous metastasis (SM) model, injection of 4T1 cells (2 × 10^5^ cells mouse^−1^) into the unilateral fat pad of the fourth mammary gland or Pan02 cells (2 × 10^5^ cells mouse^−1^) into the pancreas in situ resulted in the metastasis of 4T1 cells to the lung and Pan02 cells to the liver. For the experimental metastasis (EM) model, 2 × 10^5^ tumor cells suspended in 200 µL of PBS were injected into the tail vein, and 4T1 and Pan02 cells were subsequently transferred to the lung and liver, respectively. In the tumor resection and suture (TRS) model, 2 × 10^5^ 4T1 cells were orthotopically injected into the unilateral fat pad of the fourth mammary gland, and the tumors were surgically removed after 14 days, at which time the tumors had reached ≈200 mm^3^. The 4T1 tumors in situ and lung metastases were detected by luciferase‐based noninvasive bioluminescence imaging using the Xenogen IVIS Spectrum platform (Xenogen, USA).

The surgical protocol for generating the SM model of pancreatic cancer begins with cell preparation, wherein Pan02 cells were cultured in DMEM medium as above. Upon reaching 70–80% confluency, cells were detached using 0.25% trypsin‐EDTA, neutralized with a complete medium, and centrifuged (300 × g, 5 min). The pellet was resuspended at 2 × 10⁷ cells mL^−1^ in sterile PBS and mixed 1:1 with Matrigel to enhance engraftment efficacy. For mouse preparation, 6‐week‐old female C57BL/6 mice were fasted for 4–6 h pre‐surgery with free access to water. Anesthesia was induced via intraperitoneal injection of 2.5% tribromoethanol (300 µL/20 g body weight), supplemented by preoperative buprenorphine analgesia (0.05 mg kg^−1^, subcutaneous). Surgical execution involved positioning the mouse supine on a 37 °C heated platform, followed by rigorous sterilization: abdominal fur was removed with electric clippers, residual debris cleared with saline‐moistened gauze, and the surgical site disinfected using alternating 10% povidone‐iodine and 70% ethanol applied centrifugally over a 2.5 × 2.5 cm area, repeated twice for maximal asepsis. A 5–10 mm subcostal incision exposed the spleen and pancreatic tail, through which 20 µL of the cell‐Matrigel suspension (containing 2 × 10⁵ Pan02 cells) was injected into the pancreatic parenchyma using a 30G insulin syringe. Successful implantation was confirmed by transient fluid bleb formation. Closure entailed anatomical repositioning of organs to avoid vascular torsion, followed by layered suturing: the peritoneum was closed with 6‐0 absorbable interrupted sutures (3‐4 knots), and the skin approximated with 5‐0 nylon sutures (5‐6 stitches). Postoperative disinfection replicated the preoperative iodine‐ethanol sequence, with optional antibiotic ointment application. For postoperative care, mice recovered on a 37 °C heating pad until fully ambulatory, received sustained buprenorphine analgesia (0.05 mg kg^−1^, every 8–12 h for 48 h), and underwent daily monitoring of weight, activity, and wound integrity for 7 days. Mice exhibiting >20% weight loss or distress were euthanized per IACUC guidelines. This protocol ensured precise tumor engraftment while adhering to surgical precision and ethical postoperative standards.

### RNA‐Seq

Total RNA was isolated using TRIzol reagent (Invitrogen), and rRNA was removed using a Ribo‐Zero Gold rRNA Removal Kit (Illumina). The RNA libraries were then prepared from the rRNA‐free samples using the NEBNext Ultra II Directional RNA Library Prep Kit for Illumina (NEB) according to the manufacturer's instructions. Three independent biological replicates were carried out for each RNA‐seq experiment. The FastQC package was used to assess the sequencing quality of the raw data, and the low‐quality reads were filtered with TrimGalore. The processed data were subsequently mapped to the human reference genome (hg38) via the STAR Aligner (v2.7.3a). Count files of the aligned reads were generated by the HTSeq‐count tool. Differential expression analysis was performed with the DESeq2 package. Gene Ontology enrichment was analyzed by the R package clusterProfiler.

### RT‐qPCR

Total RNA was isolated using TRIzol (Invitrogen) and evaluated by agarose gel electrophoresis and measurement of the 260/280 and 260/230 absorbance ratios. cDNA was synthesized using HiScript II qRT SuperMix (Vazyme), and potential contaminant DNA was removed by adding DNase. Real‐time PCR was performed on a QuantStudio 3 real‐time PCR instrument using the primers shown in Table S4 (Supporting Information). All primers were purchased from Sangon Biotech and validated for specificity by amplification of serial dilutions of template cDNA. The expression of each gene was defined from the threshold cycle (Ct), and the relative levels were calculated using the 2^−^ *^ΔΔCt^* method and normalized to the levels of mRpl13a or hβ‐actin.

### shRNA Lentivirus Preparation and Infection

shRNAs were synthesized (Sangon Biotech) and inserted into the lentiviral vector pLKO.1 (Addgene). The lentiviral constructs were cotransfected with the psPAX2 packaging plasmid and the pMD2.G envelope plasmid into HEK293T cells. The viruses were collected 48 h after transfection and used to infect the cells. The shRNA sequences are shown in Table S3 (Supporting Information).

### Overexpression Experiment

For the overexpression experiments, expression plasmids encoding human Myh9 (pMYH9‐WT‐V5, Addgene plasmid 183 512), mouse Myh9 (pHalo‐MyosinIIA‐C25, Addgene plasmid 128 573), human Cyb5r3 (pHis‐3xFLAG‐CMV14‐CYB5R3, Addgene plasmid 204 551) were obtained, and their respective control plasmids from Dr. Juan Ma (Chinese Academy of Sciences). Additionally, plasmids encoding mouse Cyb5r3 (MG53013‐NM), human RPS3A (HG16468‐NF), mouse RPS3A (MG51498‐CH), and their corresponding control plasmids were purchased from Sino Biological Inc. (China). When the shRNA‐tumor cells reached 60% confluence, they were transfected with the aforementioned vectors using Lipofectamine 3000 (Thermo Fisher Scientific, Inc., USA) according to the manufacturer's instructions. After 48 h, the cells were harvested, and the overexpression efficiency was evaluated by RT‐qPCR.

### Immunofluorescence

To evaluate LD‐mediated p53 degradation, the cells were seeded on culture slides (BD Biosciences). After being fixed in 4% PFA and permeabilized in 0.5% Triton X‐100/PBS, the cells were blocked in 5% normal goat serum for 1 h at room temperature and incubated with primary antibodies overnight at 4 °C. The cells were then stained with Alexa Fluor 488‐labeled secondary antibody (Life Technologies) for 1 h at room temperature. For lipid or LD staining, the cells were incubated with Nile red (1 µm) for 30 min at 37 °C. The slides were then observed under a DeltaVision OMX V3 imaging system (Cytiva, GE Healthcare). The Fluorescence imaging was reconstructed using 3D maximum‐intensity projection in a single‐cell level. Information on the antibodies used, including their sources, catalog numbers, and dilutions, is provided in Table S5 (Supporting Information).

### DAB Staining and HE Staining

Harvested tissue samples were fixed in 4% paraformaldehyde. After 48 h, the tissues were trimmed, embedded in optimal cutting temperature medium or paraffin, and cut into 7‐µm‐thick sections for DAB staining or HE staining. Images of the stained samples were taken under an optical microscope.

### Oil Red O Staining

Oil Red O staining was performed according to a previously described protocol with slight modifications.^[^ ^55^ ^]^ Briefly, frozen tissue sections were fixed in 10% formaldehyde for 15 min. The Oil Red O working solution was prepared by mixing 6 mL of stock buffer (0.5 g of Oil Red O in 100 mL of isopropanol) with 4 mL of distilled water and then filtering through a 0.45‐µm filter. After removal of the fixative, the sections were coincubated with Oil Red O working solution for 15 min at RT, carefully washed several times with distilled water, and then observed under an optical microscope.

### Immunoprecipitation Assay

Tumor cells or purified LDs were lysed in RIPA buffer (150 mm NaCl, 0.5% sodium deoxycholate, 0.1% SDS, 1% NP‐40, 1 mm EDTA, and 50 mm Tris, pH 8.0) containing protease inhibitor cocktail for 30 min at 4 °C. After centrifugation at 12 000 × g for 30 min, the supernatants were precleared with protein A/G beads (Thermo Scientific) for 1 h and then incubated with primary antibodies overnight at 4 °C. New protein A/G beads were then added for immunoprecipitation. Precipitates were collected and detected by mass spectrometry or Western blotting with the indicated antibodies.

### Western Blot

Whole‐cell lysates were prepared in RIPA buffer at 4 °C for 30 min, and proteins were resolved by 10% SDS‒PAGE. Nuclear and cytoplasmic components were separated with a Cytoplasmic and Nuclear Fractionation Kit (Minute). Phosphorylation levels of p53 and MDM2 were assessed using co‐immunoprecipitated proteins, with total cellular p53 and MDM2 protein abundance serving as normalization controls. The proteins were then transferred to a Hybond membrane (Amersham). The membranes were blocked in 5% fat‐free milk, incubated with primary antibodies overnight at 4 °C, and then probed for 1 h with secondary horseradish peroxidase (HRP)‐conjugated anti‐mouse or anti‐rabbit IgG (antibody information is shown in Table S5, Supporting Information). After extensive washing with PBST, the target proteins were detected using enhanced chemiluminescence according to the manufacturer's instructions. β‐Actin was used as the internal control.

### Lipid Droplet Isolation

LD isolation was performed following a previously described protocol.^[^ ^29^ ^]^ Briefly, after being washed with PBS three times, the cells were scraped off with 1 mL of buffer A (20 mm tricine, 250 mm sucrose, pH 7.8, and 0.5 mm PMSF) and placed in a 15‐mL centrifuge tube, and then centrifuged at 1000 × g for 3 min at 4 °C. The cell pellets were then resuspended in 10 mL of buffer A, incubated on ice for 20 min, and disrupted using a nitrogen bomb at a pressure of 700 psi for 15 min on ice. The supernatants of the cell lysates were collected in a new 15‐mL centrifuge tube by centrifugation at 1000 × g for 3 min at 4 °C. After 2 mL of buffer B (20 mm HEPES, 100 mm KCl and 2 mm MgCl_2_, pH 7.4) was carefully placed on the top, the top layer of white material (LDs) was collected after centrifugation at 15 000 × g for 30 min at 4 °C.

### Serum Triglyceride Analysis

Mouse blood was collected by retroorbital puncture and centrifuged to fractionate the serum (15 min, 4500 rpm min^−1^), and the supernatant was collected and stored at −20 °C for later use. Serum triglyceride levels were determined using a triglyceride assay kit (Abcam, ab65336) according to the manufacturer's instructions.

### Flow Cytometry

To quantify the lipid levels in tumor cells, single cells were incubated with BODIPY (1 µm) for 30 min at 37 °C. The samples were then analyzed using FACSCalibur (BD Biosciences), and the data were analyzed using FlowJo software.

### Tumor Organoid Culture and Analysis

Tumor organoids were cultured as previously described with slight modifications.^[^ ^56^, ^57^, ^58^ ^]^ Briefly, fresh human or mouse tumor samples were stored in antibiotic‐containing DMEM supplemented with 10% FBS (PAN‐Biotech) after surgical resection and transported to the laboratory at 4 °C for immediate processing. After gentle washing at least five times with prechilled 1× DPBS, the tumor tissues were cut into small pieces with surgical scissors and digested with 2.5 mg mL^−1^ type II and type IV collagenase (Invitrogen) to obtain single‐cell suspensions. After digestion, the suspension was passed through a 40‐µm cell strainer to remove undigested material and then centrifuged at 400 × g for 5 min. The pellet was resuspended in Matrigel (Corning) and dispensed into a 24‐well cell culture dish. The Matrigel was allowed to solidify for 30 min, and then, a conditioned medium was added. The conditioned media were prepared according to previously reported protocols,^[^ ^56^, ^57^, ^58^ ^]^ with technical assistance from KINGBIO. During the first three days after passage, 10 µmol L^−1^ of the ROCK inhibitor Y‐27632 (Selleck) was added to the medium.

In some experiments, 10% serum or oleic acid (OA, 100 µm) was added to organoid cultures to promote lipid accumulation. Conversely, in some experiments, triacsin C (TC, 3 µm) was added to organoid cultures to inhibit lipid accumulation.

For routine monitoring of tumor organoid status, the Nikon ECLIPSE Ti2 inverted microscope was employed for observation and imaging. For analysis, one thousand cells from each condition were reseeded to examine organoid growth, and organoids from each group were randomly selected and quantified. To achieve this, brightfield imaging was performed using the Castor S1 system equipped (Alit Biotech, China) with a 10× objective, where 40‐layer z‐stack acquisitions were computationally fused into a single high‐resolution composite image (25 000 × 23 000 pixels, 0.46 µm/pixel scale). A deep learning‐based segmentation algorithm subsequently identified individual organoids, generating binary masks for quantitative morphometric analysis. Each mask was processed to calculate: 1) total pixel area through mask summation; 2) geometric dimensions via dual approaches – elliptical fitting (major/minor axes) and minimum bounding box extraction (X/Y diameters). All pixel‐derived measurements were converted to physical units using the predefined spatial calibration (0.46 µm//pixel scale).

### Patient‐Derived Organoid‐Based Xenograft (PDOX) Model

PDOX modeling was performed according to a previously described protocol with slight modifications.^[^ ^56^ ^]^ Briefly, patient‐derived organoids were harvested using a cell recovery solution (Corning) that could completely dissolve the BME while leaving the organoids intact. After centrifugation at 400 × g for 5 min, the organoids were resuspended in 5 mL of DPBS. After thorough mixing, 50 µL of the organoids were removed from 5 mL and disrupted into single cells using TrypLE for cell counting. Organoids (number proportionate to the number of mice) were collected and resuspended in 50% Matrigel. Six‐week‐old female NOG mice were injected subcutaneously in the right groin area with 100 µL of the mixture (5 × 10^5^ cells mouse^−1^) using a sterile 1‐mL syringe. The rate of tumor formation differed among the organoids, and tumor size was routinely monitored. Serum or 100 µm OA was injected peritumorally at a frequency of 100 µL dose^−1^ for 3 days dose^−1^ starting from the seventh day after injection to maintain a high‐lipid state of tumor cells in tumor‐bearing mice.

### In Vivo Luciferase Assays

Animals were injected intraperitoneally with 100 µL of D‐luciferin (10 mg mL^−1^) in PBS. After 10 min, the mice (under anesthesia) were imaged by means of a Xenogen IVIS Spectrum system (Xenogen, USA). After the termination of the in vivo experiments, the luciferin‐injected animals were sacrificed, and the organs were harvested and imaged within 15 min after injection.

### Patient Specimens

All participants provided informed consent before participation in the study. The studies were approved by the Ethics Committees of Ruijin Hospital affiliated with the School of Medicine, Shanghai Jiaotong University; the Ethics Committee of the 7th Medical Center of PLA General Hospital; and the Ethics Committee of the Institute of Biophysics, Chinese Academy of Sciences. Human serum was obtained from the seventh Medical Centre of PLA General Hospital. Pancreatic cancer specimens and breast cancer specimens used to prepare organoids were obtained from the Ruijin Hospital affiliated with the School of Medicine, Shanghai Jiaotong University, and the seventh Medical Centre of PLA General Hospital, respectively. Information on sources of human serum and sources of clinical samples for organoid preparations are provided in Tables S1,S2 (Supporting Information).

### Statistical Analysis

Statistical analysis was performed using GraphPad Prism software (version 5) with Student's *t*‐tests, one‐way ANOVA, two‐way ANOVA, two‐sided unpaired Wilcoxon tests, or log‐rank tests. All graphs showed the mean ± s.d. or mean ± s.e.m., as indicated in the figure legends. *P*‐values less than 0.05 were considered to indicate statistical significance. Each experiment was conducted with a minimum of three biological replicates. Mice were allocated to the experimental groups randomly.

Kaplan–Meier survival estimates for normal weight and overweight individuals in the breast cancer and pancreatic cancer groups. The 95% confidence intervals were shown in colored bands. The difference in survival between groups was evaluated with the log‐rank test (normal weight: 18.5 kg m^−^^2^ < BMI < 25 kg m^−^^2^; overweight: BMI ≥ 25 kg m^−^^2^).

Multivariate Cox regression analyzed of BMI and patient survival in the breast cancer cohort (overweight [HR: 3.08; 95% CI: 1.73–5.50]) and the pancreatic cancer cohort (overweight [HR: 1.26; 95% CI: 1.04–1.52]). (normal weight: 18.5 kg m^−^^2^ < BMI < 25 kg m^−^^2^; overweight: BMI ≥ 25 kg m^−^^2^; underweight: BMI < 18.5 kg m^−^^2^; DM: diabetes mellitus; TG: triglycerides).

## Conflict of Interest

The authors declare no conflict of interest.

## Author Contributions

H.L., L.J., Y.L., J.Z., X.C. and S.L. contributed equally to this work.H.L., H.D., L.J., X.Y., and B.S. conceptualized the study, designed the experiments, and wrote the manuscript. H.L., L.J., and H.D. performed most of the experiments with assistance from L.J., J.D., and W.Z. in collecting patient samples and clinical information, from Y.L., S.Y., and X.C. with the bioinformatics analysis, from J.Z. in preparing the gene knockdown cell lines, from X.J. with fluorescence imaging, from N.W. and P.N. with DAB, HE and fluorescence staining, from J.L., F.K., S.Y., and Z.C. with tumor organoid isolation and cultivation, and from S.L. and Y.H. in the animal experiments.

## Supporting information



Supporting Information

## Data Availability

The data that support the findings of this study are available from the corresponding author upon reasonable request.
