# Supplementary material for: Lipid Droplet‐Organized MDM2‐Mediated P53 Degradation: A Metabolic Switch Governing Diet‐Driven Tumor Progression
Source: Adv Sci (Weinh). 2025 Jun 5;12(32):e03473. doi: 10.1002/advs.202503473 (PMC12407345; doi:10.1002/advs.202503473)
Supplement: Supplementary file 1 — Supporting Information [file ADVS-12-e03473-s001.docx]

**Supporting Information**

**Lipid Droplet-Organized MDM2-Mediated P53 Degradation: A Metabolic Switch Governing Diet-Driven Tumor Progression**

Haiyang Liu^1,2,3*^, Lin Jing^4,5^, Yixin Li^6,7^, Jinxing Zhou^8^, Xiaohui Cui^9,10^, Sen Li^11^, Shijie Yang^9,10^, Fangming Kan^6^, Junfeng Du^12,13,14^, Wentao Zhong^15^, Sheng Yu^6^, Ning Wang^6^, Xing Jia^16^, Junhui Li^6^, Pan Nie^6^, Zhenzhong Chen^6^, Ying Han^6^, Lingxi Jiang^1,2,3*^, Xiyun Yan^4,5,6*^, Hongxia Duan^4,5*^, Baiyong Shen^1,2,3*^

^1^Department of General Surgery, Pancreatic Disease Center, Ruijin Hospital, Shanghai Jiao Tong University School of Medicine, Shanghai, China.

^2^Research Institute of Pancreatic Diseases, Shanghai Key Laboratory of Translational Research for Pancreatic Neoplasms, Shanghai Jiao Tong University School of Medicine, Shanghai, China.

^3^State Key Laboratory of Systems Medicine for Cancer, Institute of Translational Medicine, Shanghai Jiao Tong University, Shanghai, China.

^4^CAS Engineering Laboratory for Nanozyme, Key Laboratory of Biomacromolecules (CAS), CAS Center for Excellence in Biomacromolecules, Institute of Biophysics, Chinese Academy of Sciences, Beijing 100101, PR China.

^5^Nanozyme Laboratory in Zhongyuan, Henan Academy of Innovations in Medical Science, Zhengzhou 451163, China.

^6^University of Chinese Academy of Sciences, Beijing 100049, China.

^7^National Laboratory of Macromolecules, Institute of Biophysics, Chinese Academy of Sciences, Beijing 100101, China.

^8^National Institute of Biological Sciences, Beijing 102206, China.

^9^School of Information Science and Technology, Beijing Forestry University, Beijing, China.

^10^Engineering Research Center for Forestry-oriented Intelligent Information Processing of National Forestry and Grassland Administration, Beijing, China.

^11^Department of Biochemistry & Immunology, Capital Institute of Pediatrics, Beijing 100020, China.

^12^Medical Department of General Surgery, The 1st Medical Center, Chinese PLA General Hospital, Beijing, 100853 China.

^13^Department of General Surgery, The 7th Medical Center, Chinese PLA General Hospital, Beijing, 100700 China.

^14^The Second School of Clinical Medicine, Southern Medical University, Guangdong, 510515 China.

^15^The Second School of Clinical Medicine, Southern Medical University, Guangzhou 510515, China.

^16^Center for Biological Imaging, Core Facilities for Protein Science, Institute of Biophysics, CAS, Beijing, China.

*** Correspondence:** Baiyong Shen (shenby@shsmu.edu.cn), Hongxia Duan [(cherryshoen@aliyun.com),](mailto:(cherryshoen@ibp.ac.cn),) Xiyun Yan (yanxy@ibp.ac.cn), Lingxi Jiang [(jlx12120@rjh.com.cn)](mailto:(jlx12120@rjh.com.cn)), Haiyang Liu (haiyang_1987@126.com)

**This PDF file includes:**

Figures. S1 to S7

Tables S1 to S5

**1.** **Supplementary Figures**


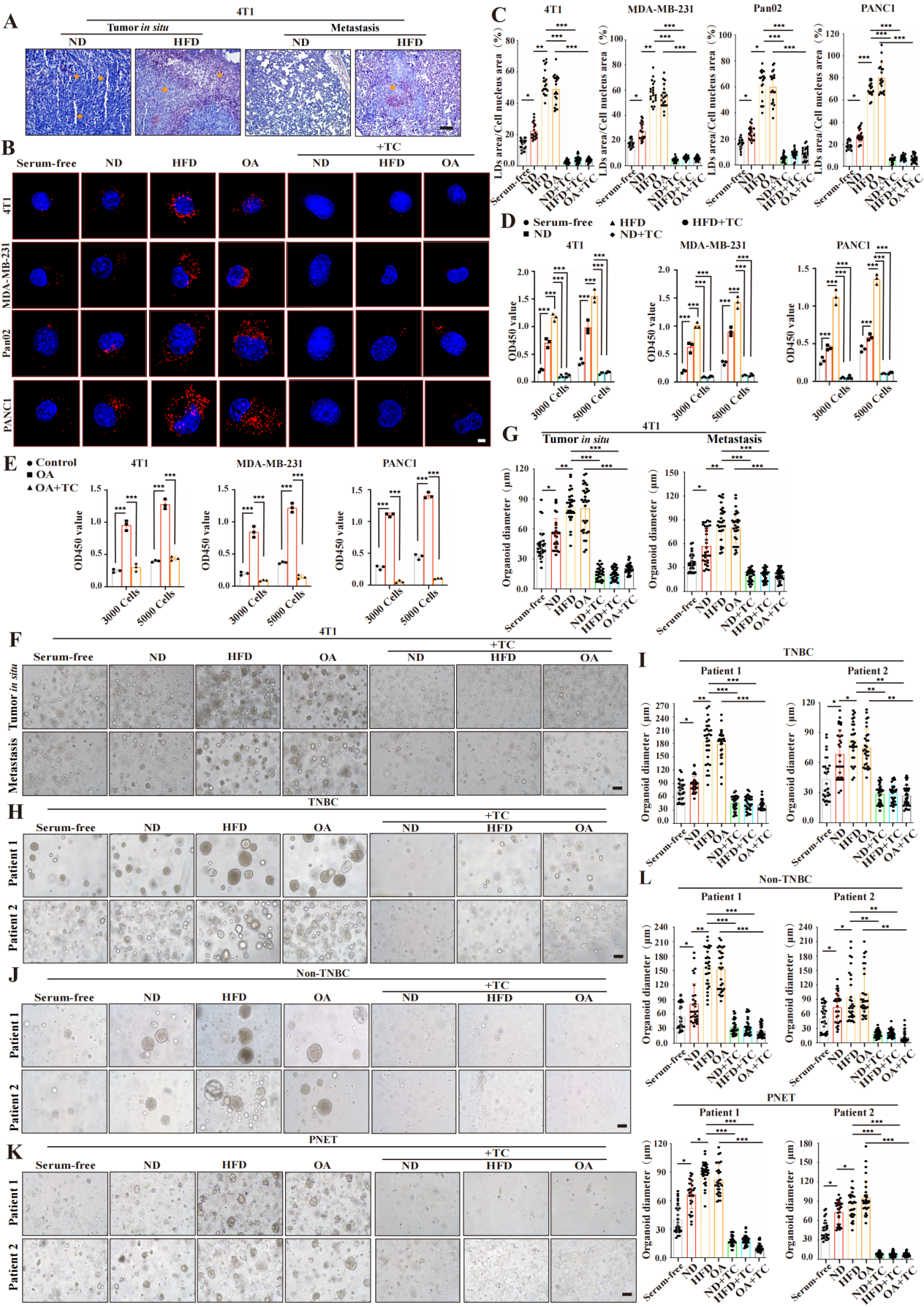


**Figure S1. Intracellular lipid drops promote tumor growth.**

**A.** Oil Red O staining reveals lipid accumulation (red) in tumor tissues, indicated by yellow stars. Groups: ND (normal diet), HFD (high-fat diet); **B-C.** Fluorescence imaging (**B**) and quantification (**C**) of lipid droplets in tumor cells under various conditions: serum-free, 10% serum with or without TC, and OA with or without TC. Conditions: serum-free (no serum), ND (serum from normal diet mouse or healthy BMI patients), HFD (serum from high-fat diet mouse or overweight patients), HFD+TC (HFD serum+TC), OA, OA+TC. **D.** Tumor cell proliferation in serum-free or 10% serum media±TC, measured by a CCK8 assay. Fetal bovine serum replaced with mouse/human serum. Conditions: serum-free (no serum), ND (serum from normal diet mouse or healthy BMI patients), HFD (serum from high-fat diet mouse or overweight patients), HFD+TC (HFD serum+TC); **E.** Proliferation of tumor cells treated with OA or TC, quantified by CCK-8 assay. Conditions: Control (fetal bovine serum), OA, OA+TC.; **F-G.** Images (**F**) and quantification (**G**) of 4T1 organoids from breast cancer tumors and liver metastases, cultured with or without 10% serum±TC or in OA media±TC. Conditions: serum-free (no serum), ND (serum from normal diet mouse), HFD (serum from high-fat diet mouse), HFD+TC (HFD serum+TC), OA, OA+TC; **H-I.** Images (**H**) and quantification (**I**) of TNBC organoids from breast cancer patients, cultured with or without 10% serum±TC or in OA media±TC. Conditions: serum-free (no serum), ND (serum from healthy BMI patients), HFD (serum from overweight patients), HFD+TC (HFD serum+TC), OA, OA+TC; **J-K.** Images of Non-TNBC (**J**) and PNET (**K**) organoids from cancer patients, cultured with or without 10% serum±TC or in OA media±TC. Conditions: serum-free (no serum), ND (serum from healthy BMI patients), HFD (serum from overweight patients), HFD+TC (HFD serum+TC), OA, OA+TC; **L.** Quantification of tumor organoids from Non-TNBC and PNET patients, cultured with or with out 10% serum±TC or in OA media±TC. Experimental conditions: mouse serum for 4T1 and Pan02; patient serum for MDA-MB-231 and PANC1. Human ND serum from healthy BMI patients and human HFD serum from overweight patients; mouse ND serum from normal diet groups and mouse HFD serum from high-fat diet groups; and 100 μM OA or 3 μM TC. Error bars: the s.d. in **D** and **E** (n=3 per group), s.e.m. in **C**, **G**, **I**, and **L** (n=30 per group). Statistical analysis: one-way ANOVA with post hoc tests for **C**, **D**, **E**, **G**, **I**, and **L**. Significance: ***, *p* < 0.001; **, *p* < 0.01; *, *p* < 0.05. Scale bars: 100 μm (**A**, **F**, **H**, **J** and **K**); 5 μm (**B**). OA: oleic acid; TC: triacsin C; TNBC: triple-negative breast cancer; Non-TNBC: non-triple-negative breast cancer; PNET: pancreatic neuroendocrine tumor.


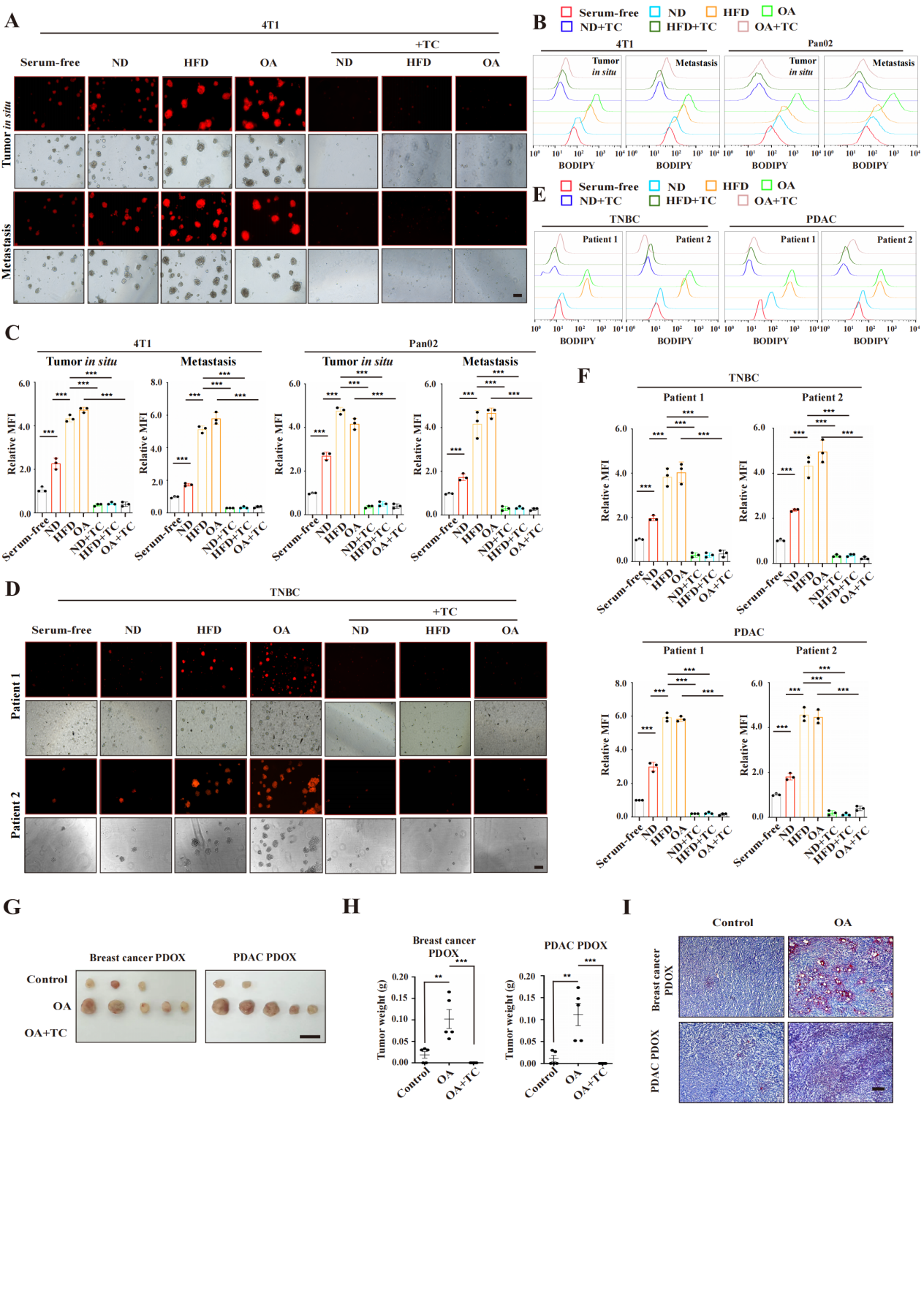


**Figure S2. Intracellular lipid drops stimulate growth of tumor cells, organoids, and PDOX models.**

**A.** Lipid accumulation in 4T1 organoids from breast cancer tumors and lung metastases, cultured with or without 10% serum±TC or in OA media±TC. Conditions: serum-free (no serum), ND (serum from normal diet mouse), HFD (serum from high-fat diet mouse), OA, ND+TC (ND serum+TC), HFD+TC (HFD serum+TC), OA+TC; **B-C.** Flow cytometry histograms (**B**) and quantification (**C**) of lipid levels in mouse cell lines derived from organoids, using BODIPY staining. Conditions as in (**A**); **D.** Lipid accumulation in TNBC organoids from patients, cultured with or without 10% serum±TC or in OA media±TC. Conditions: serum-free (no serum), ND (serum from healthy BMI patients), HFD (serum from overweight patients), OA, ND+TC (ND serum+TC), HFD+TC (HFD serum+TC), OA+TC; **E-F.** Flow cytometry histograms (**E**) and quantification (**F**) of lipid levels in patient-derived organoids from TNBC and PDAC, using BODIPY staining. Conditions as in (**D**); **G-H.** Images (**G**) and tumor weights (**H**) of PDOX tumors from breast cancer or PDAC organoids after OA stimulation±TC in NOG mice. Conditions: Control (fetal bovine serum), OA, OA+TC; **I.** Oil Red O staining of lipid accumulation (red) in PDOX tumor tissues. Conditions as in (**H**). Experimental conditions: mouse serum for 4T1 and Pan02; patient serum for MDA-MB-231 and PANC1. Human ND serum from healthy BMI patients and human HFD serum from overweight patients; and 100 μM OA or 3 μM TC. Error bars: the s.d. in **C** and **F** (n=3 per group), s.e.m. in **H** (n=5 per group). Statistical analysis: one-way ANOVA with post hoc test for **C**, **F**, and **H**. Significance: ***, *p* < 0.001; **, *p* < 0.01. Scale bars: 100 μm (**A**, **D** and **I**); 1 cm (**G**). Lipids stained with Nile red. OA: oleic acid; TC: triacsin C; TNBC: triple-negative breast cancer; PDAC: pancreatic ductal adenocarcinoma; PDOX: patient-derived organoid xenograft.


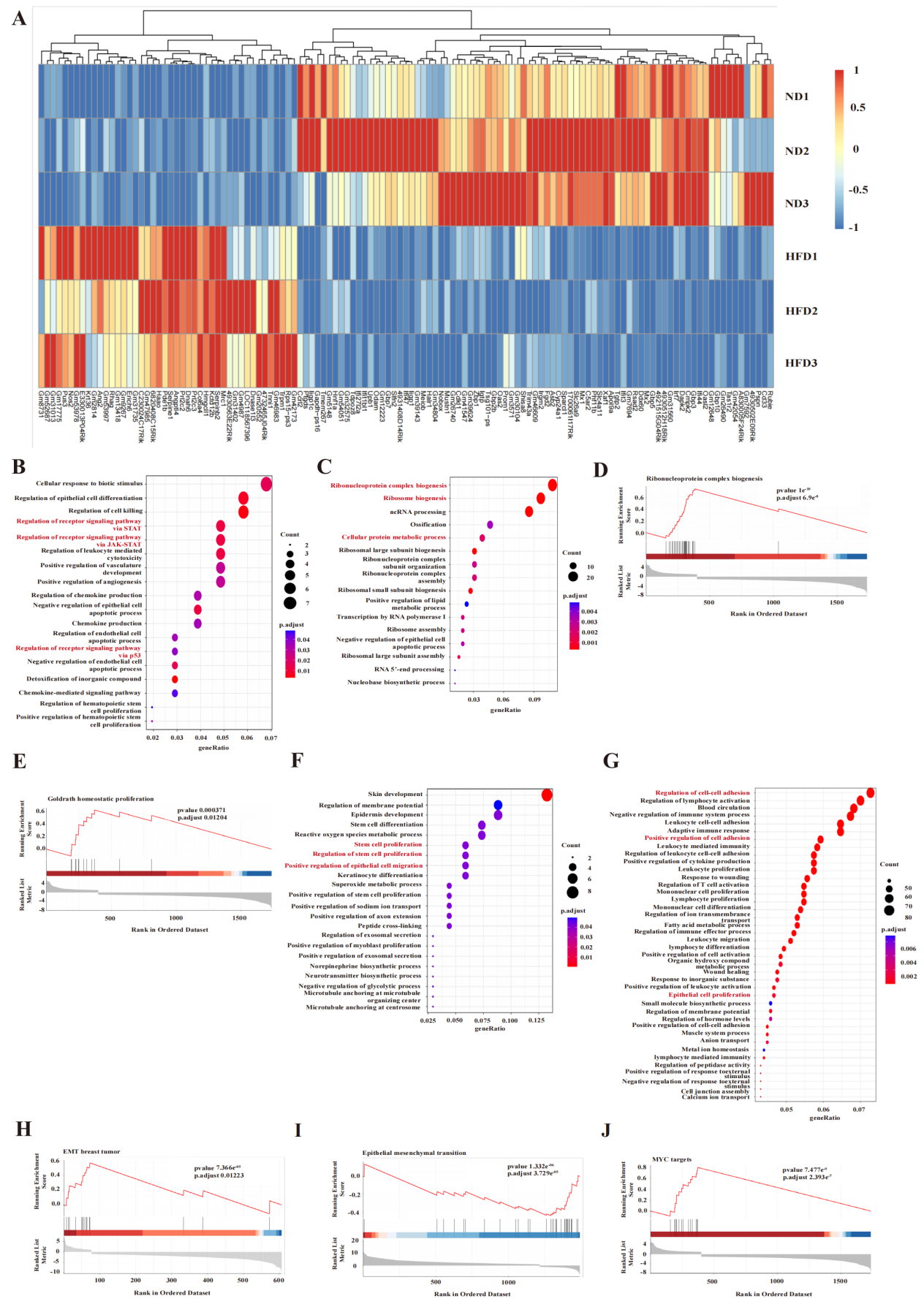


**Figure S3. Tumor cell lipid drops promote p53 degradation.**

**A.** Heatmap of differentially expressed genes (DEGs) in 4T1 cells stimulated with normal diet (ND) serum versus high-fat diet (HFD) serum; **B.** Gene Ontology analysis of DEGs in 4T1 cells treated with ND serum or HFD serum, emphasizing pathways involved in p53 and cell proliferation (red text); **C.** Gene Ontology analysis of DEGs in 4T1 cells treated with or without oleic acid, focusing on pathways related to cell proliferation and protein metabolism (red text); **D-E.** Gene set enrichment analysis for DEGs in 4T1 cells treated with or without oleic acid, focusing on pathways related to cell proliferation; **F.** Gene Ontology analysis of DEGs in 4T1 *in situ* tumors comparing ND and HFD groups, with red text indicating pathways that promote cell proliferation; **G.** Gene Ontology analysis of DEGs in *in situ* tumors and lung metastases of the 4T1 HFD group, highlighting pathways involved in cell metastasis and proliferation (red text); **H.** Gene set enrichment analysis for DEGs in 4T1 *in situ* tumors from ND and HFD groups, with a focus on epithelial-mesenchymal transition (EMT); **I.** Gene set enrichment analysis for DEGs in 4T1 *in situ* tumors and lung metastases from the HFD group; **J.** Gene set enrichment analysis comparing DEGs in 4T1 *in situ* tumors from ND and HFD groups. Experimental conditions: RNA sequencing was performed on 4T1 cells stimulated with ND or HFD serum, on 4T1 cells treated with or without oleic acid, and on *in situ* tumors and lung metastases.

**
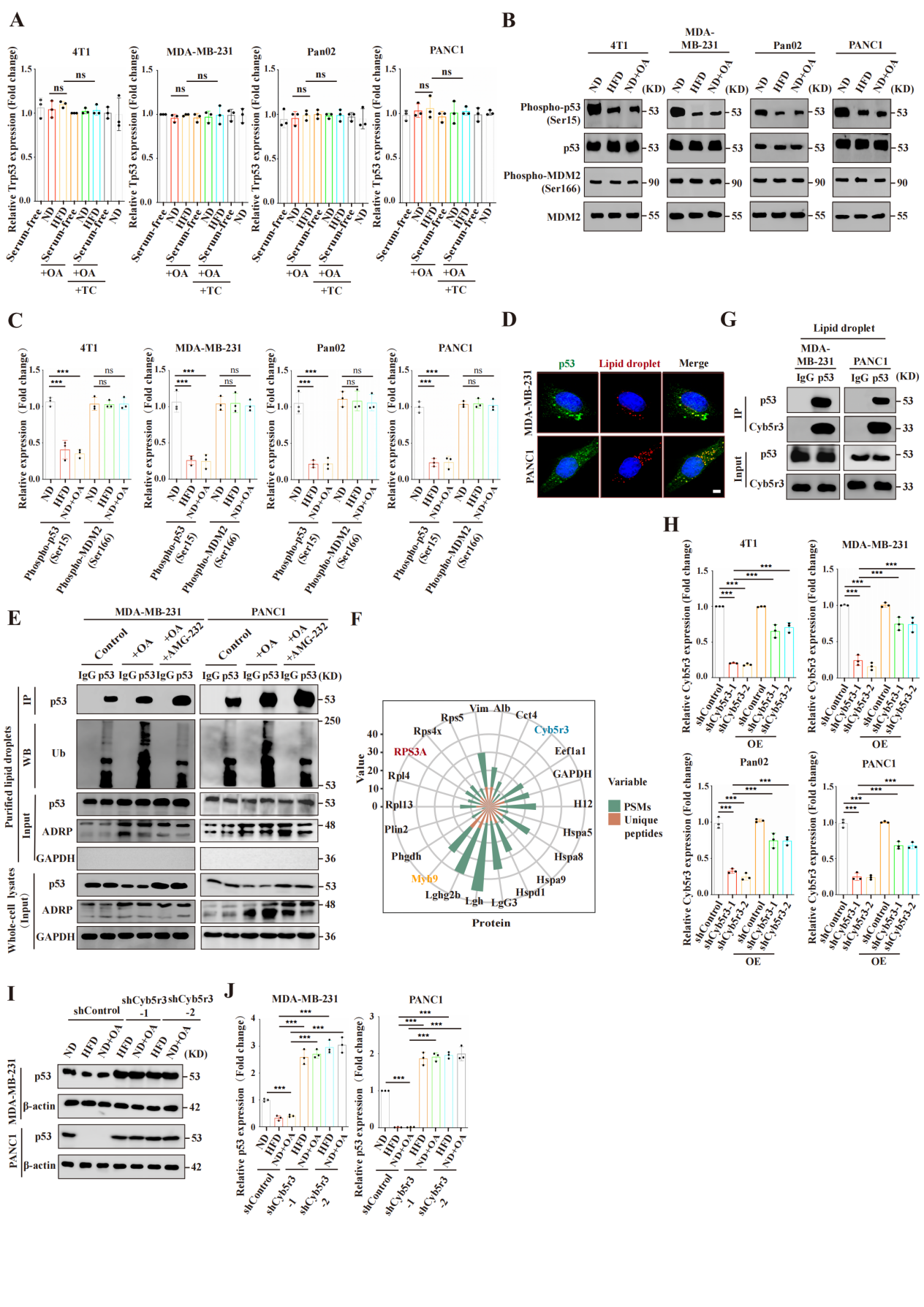
**

**Figure S4. Lipid droplets enhance MDM2-mediated p53 degradation via the Cyb5r3-Myh9 interaction.**

**A.** RT-qPCR analysis of Trp53 or Tp53 mRNA levels in tumor cells cultured with or without 10% serum with or without TC, and in OA media with or without TC. Conditions: serum-free (no serum), ND (serum from normal diet mouse or healthy BMI patients), HFD (serum from high-fat diet mouse or overweight patients); **B-C.** Western blot analysis (**B**) and quantification (**C**) of p53 and MDM2 phosphorylation levels in tumor cells. Proteins isolated via Co-Immunoprecipitation (Co-IP) were analyzed for phosphorylation status, with total p53 and total MDM2 levels serving as loading controls. Conditions: ND (serum from normal diet mouse or healthy BMI patients), HFD (serum from high-fat diet mouse or overweight patients), ND+TC (ND serum+TC); **D.** Fluorescence staining illustrates p53 localization on LDs; **E.** Immunoprecipitation and Western blotting analysis of MDM2-mediated p53 ubiquitination (Ub) on LDs. LDs were purified from an equal number of control, OA-treated and OA+AMG-232-treated tumor cells. AMG-232 inhibits MDM2 activity, with ADRP and GAPDH as LD and cytosolic fraction markers, respectively. Control: fetal bovine serum; **F.** Mass spectrometry identified potential p53-interacting proteins involved in LD-mediated ubiquitination; **G.** Immunoprecipitation and Western blotting analysis demonstrated the interaction between p53 and Cyb5r3 on purified LDs from tumor cells; **H.** RT-qPCR confirms the knockdown and overexpression (OE) efficiency of Cyb5r3 in tumor cells; **I-J.** Western blot (**I**) and quantification (**J**) show the effect of Cyb5r3 knockdown (shCyb5r3-1 and shCyb5r3-2) on p53 levels in tumor cells. Conditions: ND (serum from healthy BMI patients), HFD (serum from overweight patients). LDs were visualized using Nile red staining. Experimental conditions: mouse serum for 4T1 and Pan02; patient serum for MDA-MB-231 and PANC1. Human ND serum from healthy BMI patients and human HFD serum from overweight patients; and 100 μM OA, 3 μM TC or 4 μM AMG-232. β-actin was used as a control for Western blotting. Error bars: the s.d. in **A**, **C**, **H** and **J** (n=3 per group). Statistical analysis: one-way ANOVA with post hoc test for **A**, **C**, **H** and **J**. Significance: ***, *p* < 0.001; ns, *p*>0.05. Scale bars: 5 μm (**D**). LD: lipid droplet; OA: oleic acid; TC: triacsin C.


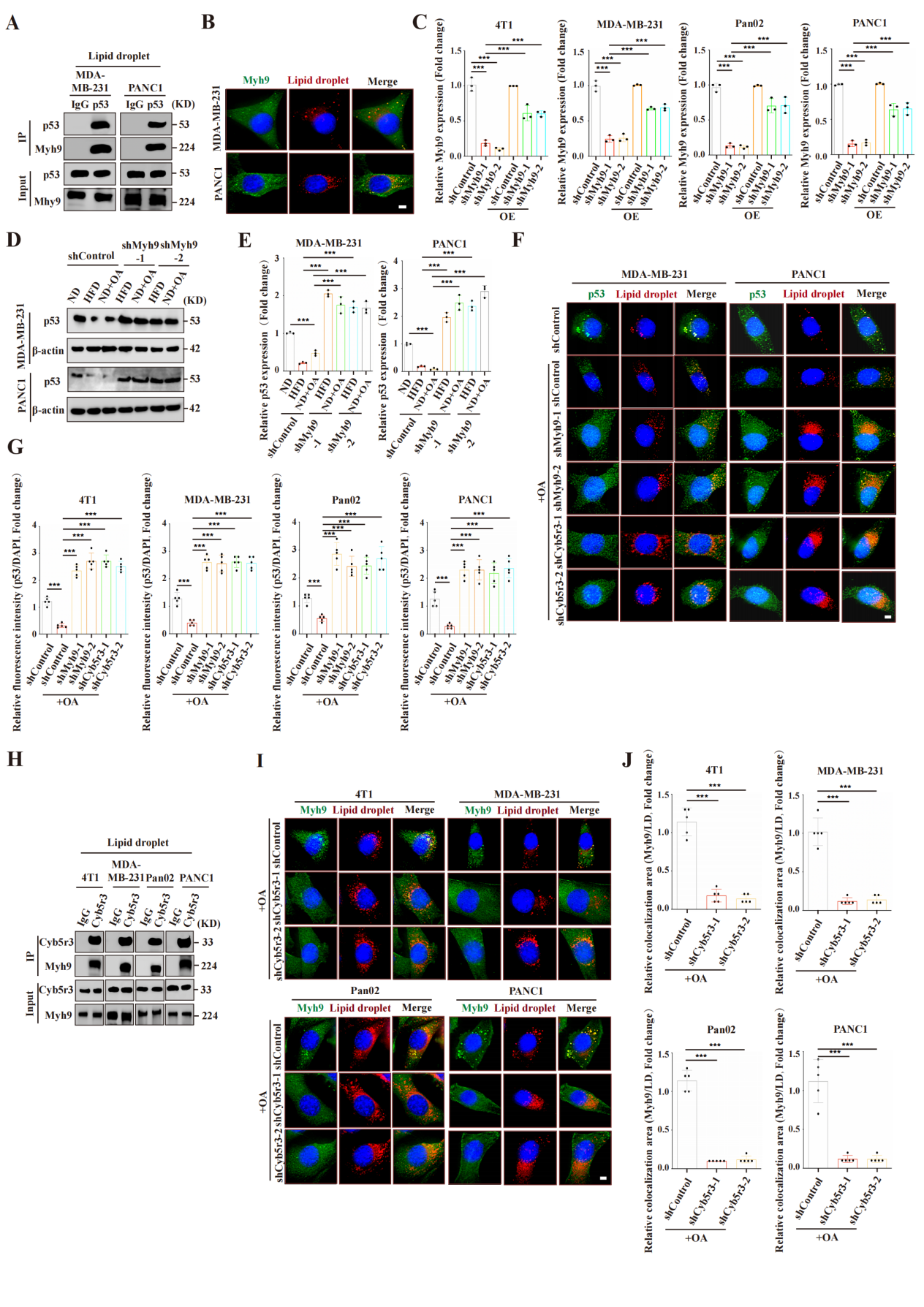


**Figure S5. Lipid droplets enhance MDM2-mediated p53 degradation via the Cyb5r3-Myh9 interaction.**

**A.** Immunoprecipitation and Western blotting analysis revealed the interaction between p53 and Myh9 on purified LDs from tumor cells; **B.** Fluorescence staining demonstrates Myh9 localization on LDs; **C.** RT-qPCR confirms the knockdown and overexpression (OE) efficiency of Myh9 in tumor cells; **D-E.** Western blot (**D**) and quantification (**E**) show the impact of Myh9 knockdown (shMyh9-1 and shMyh9-2) on p53 levels in tumor cells. Conditions: ND (serum from healthy BMI patients), HFD (serum from overweight patients), ND+OA (ND serum+OA); **F-G.** Fluorescence staining (**F**) and quantification (**G**) illustrate the effects of Myh9 and Cyb5r3 knockdown on p53 localization to LDs in OA-treated tumor cells; **H.** Immunoprecipitation and Western blotting demonstrate the interaction between Cyb5r3 and Myh9 on purified LDs from tumor cells; **I-J.** Fluorescence staining (**I**) and quantification (**J**) show the effect of Cyb5r3 knockdown on Myh9 localization to LDs in OA-treated tumor cells. LDs were visualized using Nile red staining. Experimental conditions: patient serum for MDA-MB-231 and PANC1. Human ND serum from healthy BMI patients and human HFD serum from overweight patients. 100 μM OA. β-actin was used as a control for Western blotting. Error bars: the s.d. in **C** and **E** (n=3 per group), s.e.m. in **G** and **J** (n=5 per group). Statistical analysis: one-way ANOVA with post hoc test for **C**, **E**, **G** and **J**. Significance: ***, *p* < 0.001. Scale bars: 5 μm (**B**, **F** and **I**). LD: lipid droplet; OA: oleic acid.

**
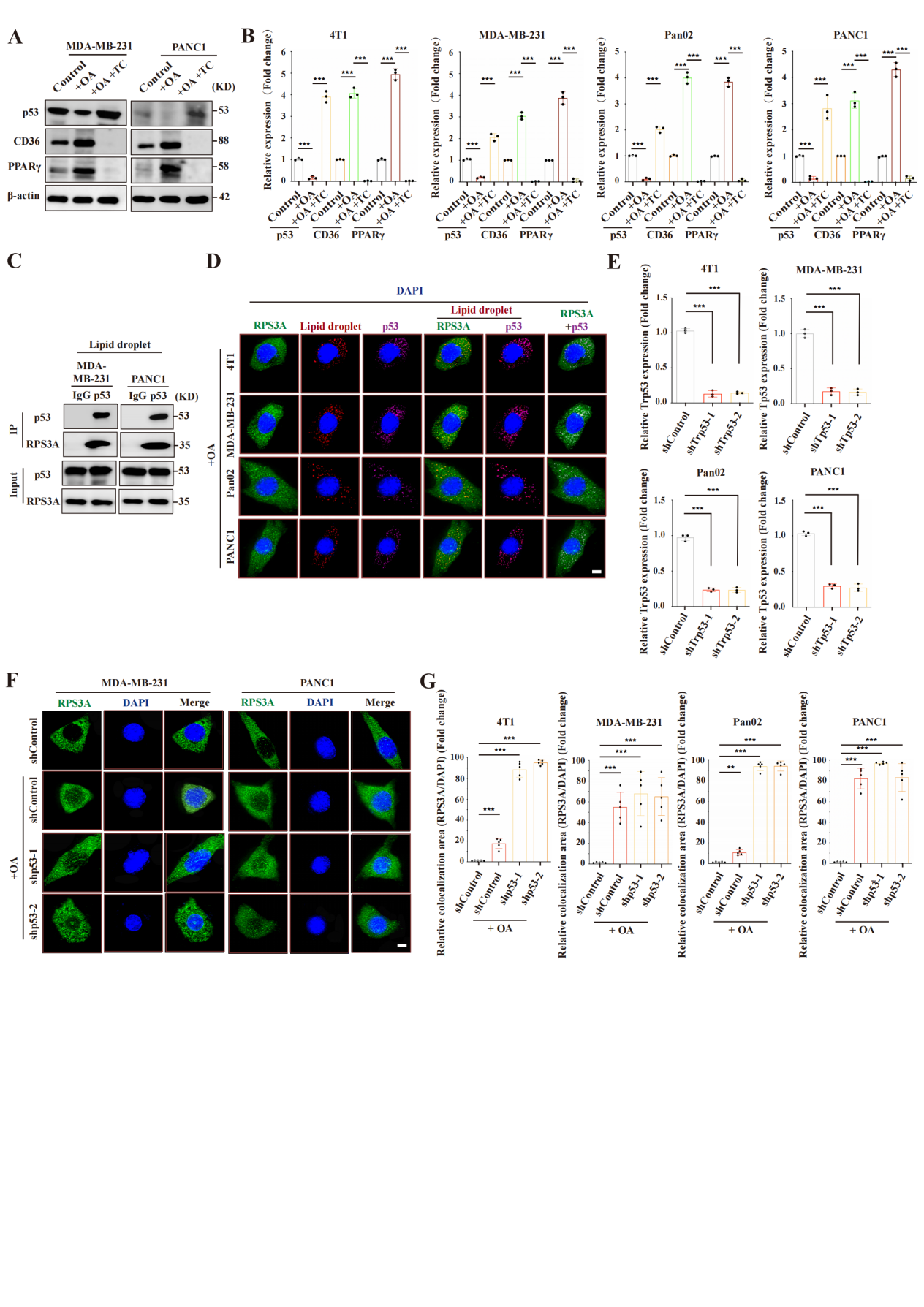
**

**Figure S6. p53 degradation enhances intracellular lipid accumulation by regulating the nuclear import of the RPS3A-C/EBPβ complex.**

**A-B.** Western blotting analysis (**A**) and quantification (**B**) of p53, CD36 and PPARγ expression in tumor cells treated with or without OA or TC; **C.** Immunoprecipitation and Western blotting analysis demonstrated the interaction between p53 and RPS3A on purified LDs from tumor cells; **D.** Fluorescence staining confirms colocalization of RPS3A, p53, and LDs in tumor cells; **E.** RT-qPCR assesses the knockdown efficiency of Trp53 or Tp53 in tumor cells; **F-G.** Fluorescence staining (**F**) and quantification (**G**) show the effect of p53 knockdown on RPS3A nuclear localization in OA-treated tumor cells. Experimental conditions: 100 μM OA or 3 μM TC. Control conditions used fetal bovine serum. Error bars: s.d. in **B** and **E** (n=3 per group); s.e.m. in **G** (n=5 per group). Statistical analysis: one-way ANOVA with post hoc test for **E** and **G;** two-way ANOVA with post hoc test for **B**. Significance: ***, *p* < 0.001; **, *p* < 0.01. Scale bars: 5 μm (**D** and **F**). Lipids stained with Nile red. β-actin was used as a control for Western blotting. Control: fetal bovine serum; OA: oleic acid; TC: triacsin C.


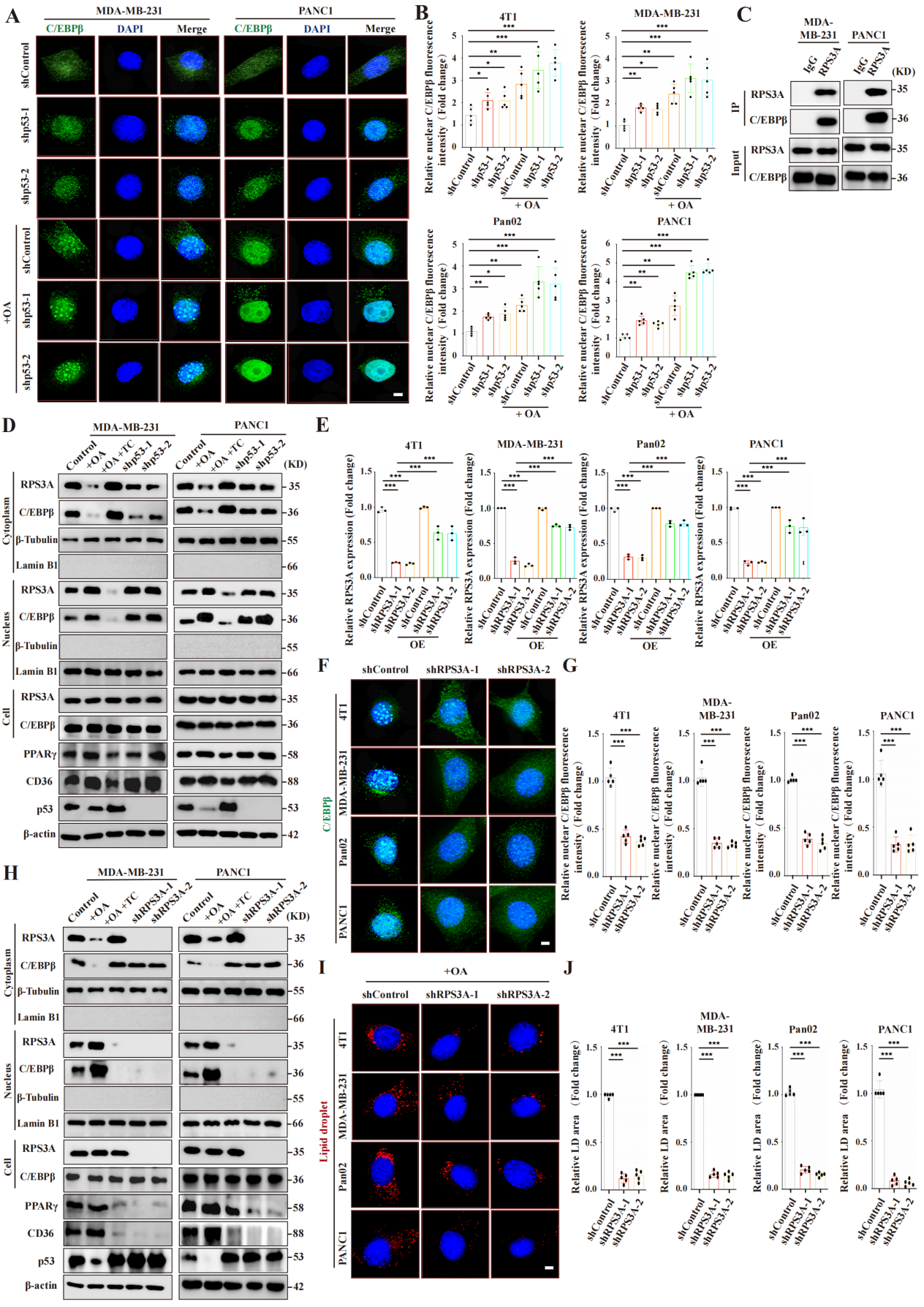


**Figure S7. p53 degradation enhances intracellular lipid accumulation by regulating nuclear import of the RPS3A-C/EBPβ Complex.**

**A-B.** Fluorescence staining (**A**) and quantification (**B**) illustrate the impact of p53 knockdown (shp53-1 and shp53-2) on C/EBPβ nuclear localization in tumor cells treated with or without OA; **C.** Immunoprecipitation and Western blotting showing the interaction between RPS3A and C/EBPβ in tumor cells; **D.** Western blotting analysis of nuclear and cytoplasmic fractions shows the effect of p53 knockdown on RPS3A and C/EBPβ localization in tumor cells treated with or without OA or TC; **E.** RT-qPCR assesses RPS3A knockdown and overexpression (OE) efficiency in tumor cells; **F-G.** Fluorescence imaging (**F**) and quantification (**G**) demonstrate the effect of RPS3A knockdown (shRPS3A-1 and shRPS3A-2) on C/EBPβ nuclear localization in tumor cells; **H.** Western blotting analysis of nuclear and cytoplasmic fractions shows the effect of RPS3A knockdown on RPS3A and C/EBPβ localization in tumor cells treated with or without OA or TC; **I-J.** Fluorescence imaging (**I**) and quantification (**J**) show the influence of RPS3A knockdown on LD accumulation in OA-treated tumor cells. Experimental conditions: 100 μM OA or 3 μM TC. Control conditions used fetal bovine serum. Error bars: s.d. in **E** (n=3 per group); s.e.m. in **B**, **G** and **J** (n=5 per group). Statistical analysis: one-way ANOVA with post hoc test for **B**, **E**, **G** and **J**. Significance: ***, *p* < 0.001; **, *p* < 0.01; *, *p* < 0.05. Scale bars: 5 μm (**A**, **F** and **I**). Lipids stained with Nile red. β-actin was used as a control for Western blotting. Control: fetal bovine serum; OA: oleic acid; TC: triacsin C.


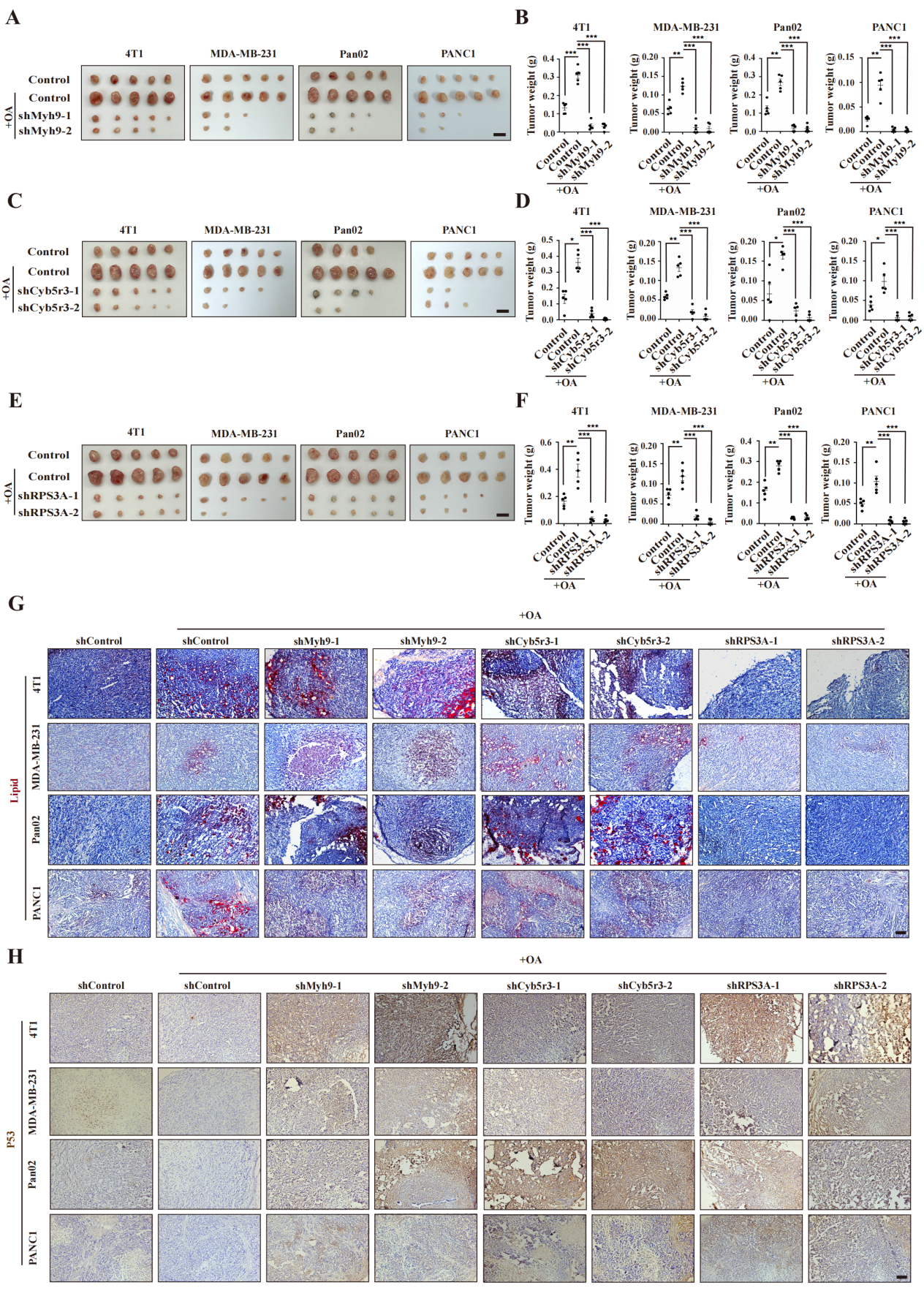


**Figure S8. The lipid droplet-p53 regulatory circuit enhances tumor growth.**

**A-B.** Photographs (**A**) and tumor weights (**B**) of xenograft tumors derived from control, OA-treated, and OA-treated Myh9-knockdown (shMyh9-1 and shMyh9-2) tumor cells; **C-D.** Photographs (**C**) and tumor weights (**D**) of xenograft tumors derived from control, OA-treated, and Cyb5r3-knockdown (shCyb5r3-1 and shCyb5r3-2) tumor cells; **E-F.** Photographs (**E**) and tumor weights (**F**) of xenograft tumors derived from control, OA-treated, and RPS3A-knockdown (shRPS3A-1 and shRPS3A-2) tumor cells; **G-H.** Representative Oil Red O staining (**G**) and DAB staining (**H**) showing lipid accumulation (red) and p53 accumulation in tumor tissues. Experimental conditions: tumor cells were treated with fetal bovine serum (control) or 100 μM OA. Error bars: s.e.m. in **B**, **D** and **F** (n=5 per group). Statistical analysis: one-way ANOVA with post hoc test for **B**, **D** and **F**. Significance: ***, *p* < 0.001; **, *p* < 0.01; *, *p* < 0.05. Scale bars: 100 μm (**G** and **H**); 1 cm (**A**, **C** and **E**). OA: oleic acid.


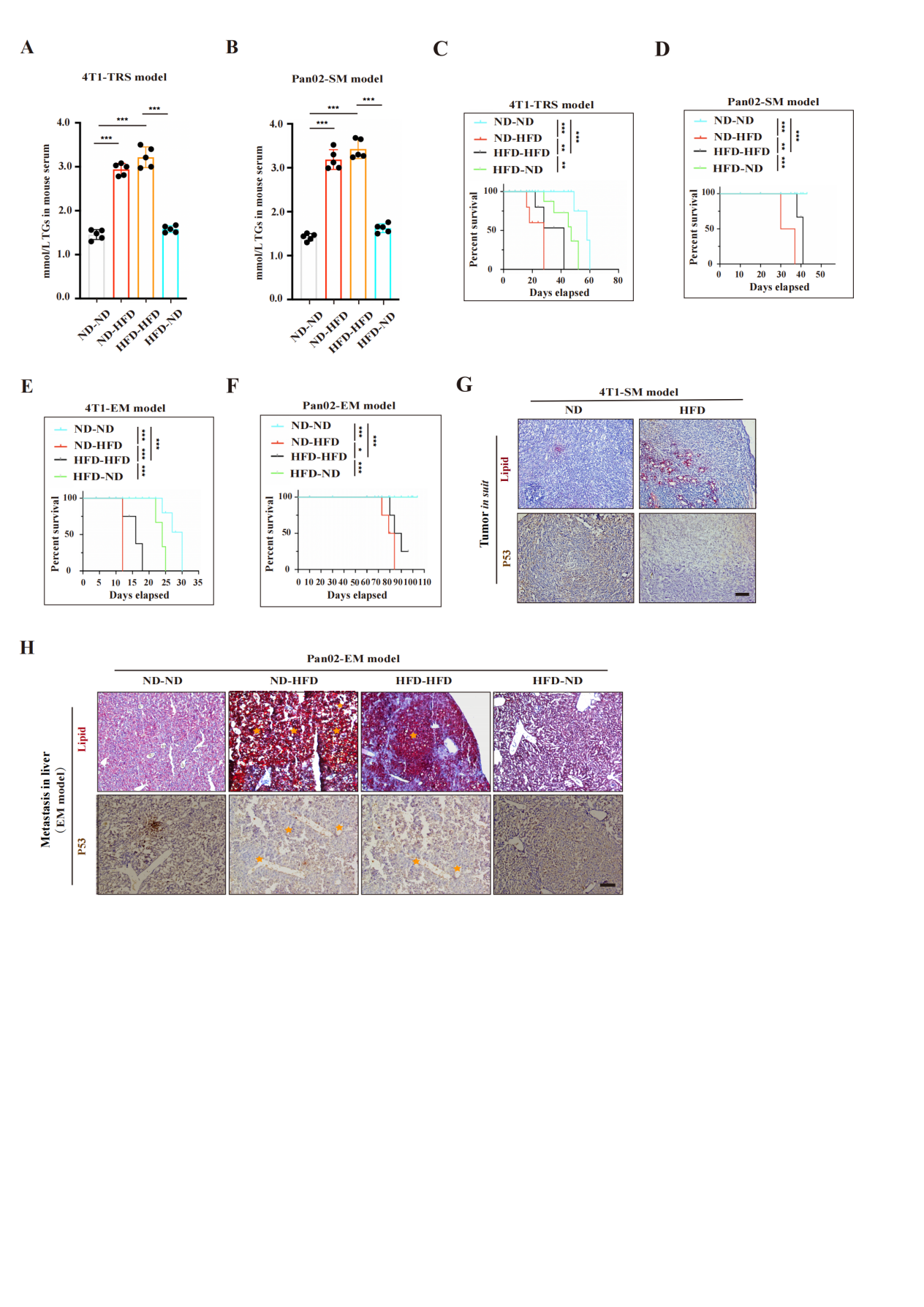


**Figure S9. Lipid restriction impedes tumor growth.**

**A.** Serum triglyceride levels in 4T1 tumor-bearing mice under different dietary conditions; **B.** Serum triglyceride levels in Pan02 tumor-bearing mice under varied diets; **C-F.** Survival curves for mice with dietary changes in the 4T1 TRS (**C**), Pan02 SM (**D**), 4T1 EM (**E**), and Pan02 EM (**F**) models; **G.** Oil Red O staining (upper) and DAB staining (lower) showing lipid accumulation (red) and p53 accumulation in 4T1 SM model tumor tissues; **H.** Oil Red O and DAB staining indicating lipid accumulation (red) and p53 presence in Pan02 EM model tumors (yellow stars). Experimental conditions: mice were fed either a normal diet (ND) or a high-fat diet (HFD). Error bars: s.e.m. in **A** and **B** (n=5 per group). Statistical analysis: one-way ANOVA with post hoc test for **A** and **B**; log-rank test for **C-F**. Significance: ***, *p* < 0.001; **, *p* < 0.01; *, *p* < 0.05. Scale bars: 100 μm (**G** and **H**). TRS: tumor resection and suture; SM: spontaneous metastasis; EM: experimental metastasis.

1. **Supplementary Tables**

**Table S1. Serum triglycerides in breast cancer patients.**

| **Number** | **Patient ID** | **Sex** | **Age** | **BMI** | **Triglycerides**  **（TG）（mmol/）** | **Group** |
| --- | --- | --- | --- | --- | --- | --- |
| P1 | 23240A | Female | 49 | 28.8 | 4.36 | Hypertriglyceridemia |
| P2 | 17556A | Female | 67 | 25.6 | 3.81 |  |
| P3 | 07083A | Female | 58 | 27.8 | 4.06 |  |
| P4 | 05483A | Female | 66 | 28.6 | 5.08 |  |
| P5 | 04923A | Female | 42 | 26.6 | 3.19 |  |
| P6 | 998976 | Female | 59 | 27.7 | 3.19 |  |
| P7 | 984905 | Female | 50 | 25.3 | 5.09 |  |
| P8 | 981816 | Female | 58 | 25.4 | 3.24 |  |
| P9 | 980369 | Female | 67 | 25.2 | 3.32 |  |
| P10 | 979698 | Female | 41 | 25 | 3.22 |  |
| P11 | 979119 | Female | 38 | 26.2 | 3.63 |  |
| P12 | 975088 | Female | 33 | 28.4 | 4.45 |  |
| P13 | 948452 | Female | 37 | 30.1 | 7.47 |  |
| P14 | 898293 | Female | 45 | 27 | 7.62 |  |
| P15 | 891168 | Female | 56 | 25.3 | 4.95 |  |
| P16 | 27694A | Female | 58 | 22.5 | 0.94 | Normal triglycerides |
| P17 | 27292A | Female | 57 | 19 | 1.03 |  |
| P18 | 25373A | Female | 56 | 21.6 | 1.3 |  |
| P19 | 25229A | Female | 56 | 23.9 | 0.81 |  |
| P20 | 06565A | Female | 65 | 20.7 | 0.79 |  |
| P21 | 23118A | Female | 67 | 20.9 | 0.72 |  |
| P22 | 21655A | Female | 45 | 19.5 | 0.83 |  |
| P23 | 21643A | Female | 66 | 20.8 | 1.05 |  |
| P24 | 21326A | Female | 39 | 19.8 | 1.19 |  |
| P25 | 20041A | Female | 46 | 18.2 | 1.18 |  |
| P26 | 19280A | Female | 59 | 23 | 1 |  |
| P27 | 17636A | Female | 66 | 19.7 | 0.48 |  |
| P28 | 17212A | Female | 44 | 19.7 | 0.96 |  |
| P29 | 16768A | Female | 56 | 22.9 | 1.18 |  |
| P30 | 11994A | Female | 54 | 23.4 | 0.5 |  |

**Table S2. Patient information for the preparation of tumor organoids.**

| **Patient ID** | **Sex** | **Age** | **Tumor type** | **Clinical typing** | **Note** |
| --- | --- | --- | --- | --- | --- |
| RX202303001 | Female | 57 | Breast cancer | Non-TNBC (Her2^+^ER^+^PR^-^) | *In situ* tumor |
| RX202304001 | Female | 59 | Breast cancer | Non-TNBC  (Her2^+^ER^-^PR^-^) | *In situ* tumor |
| RX202305001 | Female | 50 | Breast cancer | TNBC | *In situ* tumor |
| RX202306001 | Female | 50 | Breast cancer | TNBC | Hepatic metastasis |
| Y202306007 | Female | 76 | Pancreatic cancer | PDAC | *In situ* tumor |
| Y202305005 | Female | 66 | Pancreatic cancer | PDAC | *In situ* tumor |
| PNET202309001 | Female | 70 | Pancreatic cancer | PNET | Hepatic metastasis |
| PNET202309002 | Male | 51 | Pancreatic cancer | PNET | Hepatic metastasis |

**Table S3. Sequences of shRNA.**

| **Name** | **Sense (5’-3’)** | **Antisense (5’-3’)** |
| --- | --- | --- |
| shhMyh9-1 | ccgggacagcaatctgtaccgcattctcgagaatgcggtacagattgctgtcg | aattcgacagcaatctgtaccgcattctcgagaatgcggtacagattgctgtc |
| shhMyh9-2 | ccgggccaagctcaagaacaagcatctcgagatgcttgttcttgagcttggcg | aattcgccaagctcaagaacaagcatctcgagatgcttgttcttgagcttggc |
| shmMyh9-1 | ccggcggtaaattcattcgtatcaactcgagttgatacgaatgaatttaccgg | aattccggtaaattcattcgtatcaactcgagttgatacgaatgaatttaccg |
| shmMyh9-2 | ccgggccatacaacaaataccgcttctcgagaagcggtatttgttgtatggcg | aattcgccatacaacaaataccgcttctcgagaagcggtatttgttgtatggc |
| shhCyb5r3-1 | ccggcctggtcatcaaggtttacttctcgagaagtaaaccttgatgaccaggg | aattccctggtcatcaaggtttacttctcgagaagtaaaccttgatgaccagg |
| shhCyb5r3-2 | ccggtctacctctcggctcgaattgctcgagcaattcgagccgagaggtagag | aattctctacctctcggctcgaattgctcgagcaattcgagccgagaggtaga |
| shmCyb5r3-1 | ccggcgaacattctgctcgcttcaactcgagttgaagcgagcagaatgttcgg | aattccgaacattctgctcgcttcaactcgagttgaagcgagcagaatgttcg |
| shmCyb5r3-2 | ccgggtgaagtctgtaggcatgattctcgagaatcatgcctacagacttcacg | aattcgtgaagtctgtaggcatgattctcgagaatcatgcctacagacttcac |
| shhTp53-1 | ccggcggcgcacagaggaagagaatctcgagattctcttcctctgtgcgccgg | aattccggcgcacagaggaagagaatctcgagattctcttcctctgtgcgccg |
| shhTp53-2 | ccgggtccagatgaagctcccagaactcgagttctgggagcttcatctggacg | aattcgtccagatgaagctcccagaactcgagttctgggagcttcatctggac |
| shmTrp53-1 | ccggccactacaagtacatgtgtaactcgagttacacatgtacttgtagtggg | aattcccactacaagtacatgtgtaactcgagttacacatgtacttgtagtgg |
| shmTrp53-2 | ccggctacaagaagtcacagcacatctcgagatgtgctgtgacttcttgtagg | aattcctacaagaagtcacagcacatctcgagatgtgctgtgacttcttgtag |
| shhRPS3A-1 | ccgggccaagaagaaagtggttgatctcgagatcaaccactttcttcttggcg | aattcgccaagaagaaagtggttgatctcgagatcaaccactttcttcttggc |
| shhRPS3A-2 | ccgggcccaagtttgaattgggaaactcgagtttcccaattcaaacttgggcg | aattcgcccaagtttgaattgggaaactcgagtttcccaattcaaacttgggc |
| shmRPS3A-1 | ccgggcattgggaaagacatagaaactcgagtttctatgtctttcccaatgcg | aattcgcattgggaaagacatagaaactcgagtttctatgtctttcccaatgc |
| shmRPS3A-2 | ccggagaatgatgaagttgcgtttactcgagtaaacgcaacttcatcattctg | aattcagaatgatgaagttgcgtttactcgagtaaacgcaacttcatcattct |

**Table S4. Sequences of qPCR primers.**

| **Name** | **sense (5’-3’)** | **antisense (5’-3’)** |
| --- | --- | --- |
| hMyh9 | agagctcacgtgcctcaacg | tgaccacacagaacaggcctg |
| mMyh9 | tgtcattagtgtcctgagagcag | caggcatggagtttgtgatg |
| hCyb5r3 | ttgccaaccagaccgagaagga | ccagcgtgtaccagagcttgaag |
| mCyb5r3 | cagggcttcgtgaatgaggag | tccacacatcagtatcagcgg |
| hTp53 | ccctcctcagcatcttatccg | gcacaaacacgcacctcaaa |
| mTrp53 | ggcagacttttcgccacag | caggcacaaacacgaacctc |
| hRPS3A | tggatcttacccgtgacaaaatg | tgacatcaacgtgagcttcaatc |
| mRPS3A | aacaagcgcctgacgaaag | agtgtcttcccgatgttcctaat |
| hβ-actin | cctcgcctttgccgatcc | aggaatccttctgacccatgc |
| mRpl13a | gaggtcgggtggaagtacca | tgcatcttggccttttcctt |

**Table S5. Antibodies used in the various analyses conducted in this study.**

| **Antibody** | **Species** | **Dilution** | **Source** | **Identifier** |
| --- | --- | --- | --- | --- |
| Myh9 | Rabbit | 1:5000 for WB  1:100 for ICC/IF | Proteintech | 11128-1-AP |
| Cyb5r3 | Rabbit | 1:1000 | Proteintech | 10894-1-AP |
| CD36 | Rabbit | 1:500 | Invitrogen | PA1-16813 |
| PPARγ | Rabbit | 1:1000 | Proteintech | 16643-1-AP |
| C/EBPβ | Rabbit | 1:5000 for WB  1:100 for ICC/IF | Proteintech | 23431-1-AP |
| RPS3A | Rabbit | 1:1000 for WB  1:100 for ICC/IF | Proteintech | 14123-1-AP |
| Lamin B1 | Rabbit | 1:5000 | Proteintech | 12987-1-AP |
| β-Tubulin | Rabbit | 1:1000 | Proteintech | 10068-1-AP |
| Ubiquitin | Rabbit | 1:1000 | Proteintech | 10201-2-AP |
| p53 | Mouse | 1:500 for WB  1:100 for ICC/IF  1:100 for DAB | Abcam | ab26 |
| β-actin | Rabbit | 1:1000 | CST | 4967 |
| ADRP | Rabbit | 1:1000 | Abcam | ab108323 |
| GAPDH | Mouse | 1:1000 | Abcam | ab8245 |
| 488-goat anti-mouse IgG | Goat | 1:200 | Life Technologies | A-11001 |
| 488-goat anti-rabbit IgG | Goat | 1:200 | Life Technologies | A-11008 |
| HRP-goat anti-rabbit IgG | Goat | 1:6000 | EMAR | EM35111 |
| HRP-goat anti-mouse IgG | Goat | 1:6000 | EMAR | EM35110 |
